# Cell death triggering and effector recognition by Sw‐5 SD‐CNL proteins from resistant and susceptible tomato isolines to *Tomato spotted wilt virus*

**DOI:** 10.1111/mpp.12439

**Published:** 2016-08-14

**Authors:** Athos Silva De Oliveira, Ivo Koolhaas, Leonardo Silva Boiteux, Octav F. Caldararu, Andrei‐Jose Petrescu, Renato Oliveira Resende, Richard Kormelink

**Affiliations:** ^1^ Laboratory of Virology, Department of Plant Sciences Wageningen University Droevendaalsesteeg 1 Wageningen PB 6708 the Netherlands; ^2^ Department of Cell Biology, Institute of Biological Sciences University of Brasília (UnB) Asa Norte 70910‐900 Brasília DF Brazil; ^3^ Embrapa Vegetable Crops (CNPH) BR 060 Km 09 70351‐970 Brasília DF Brazil; ^4^ Department of Bioinformatics and Structural Biochemistry Institute of Biochemistry of the Romanian Academy Splaiul Independentei 296 Bucharest 060036 Romania

**Keywords:** NB‐LRR, NS_M_, SD‐CNL, Sw‐5, Sw‐5b, tomato, tospoviruses

## Abstract

Only a limited number of dominant resistance genes acting against plant viruses have been cloned, and further functional studies of these have been almost entirely limited to the resistance genes *Rx* against *Potato virus X* (PVX) and *N* against *Tobacco mosaic virus* (TMV). Recently, the cell‐to‐cell movement protein (NS_M_) of *Tomato spotted wilt virus* (TSWV) has been identified as the avirulence determinant (Avr) of Sw‐5b‐mediated resistance, a dominant resistance gene which belongs to the class of SD‐CC‐NB‐LRR (Solanaceae domain‐coiled coil‐nucleotide‐binding‐leucine‐rich repeat, SD‐CNL) resistance genes. On transient expression of the NS_M_ protein in tomato and transgenic *Nicotiana benthamiana* harbouring the *Sw‐5b* gene, a hypersensitive cell death response (HR) is triggered. Here, it is shown that high accumulation of the Sw‐5b protein in *N. benthamiana* leaves, achieved by co‐expression of the Sw‐5b protein with RNA silencing suppressors (RSSs), leads to auto‐activity in the absence of NS_M_. In a similar approach, Sw‐5a, the highest conserved paralogue of Sw‐5b from *Solanum peruvianum*, also triggered HR by auto‐activation, whereas the highest conserved orthologue from susceptible *S. lycopersicum*, named Sw‐5a^S^, did not. However, neither of the last two homologues was able to trigger an NS_M_‐dependent HR. Truncated and mutated versions of these Sw‐5 proteins revealed that the NB‐ARC [nucleotide‐binding adaptor shared by Apaf‐1 (from humans), R proteins and CED‐4 (from nematodes)] domain is sufficient for the triggering of HR and seems to be suppressed by the SD‐CC domain. Furthermore, a single mutation was sufficient to restore auto‐activity within the NB‐ARC domain of Sw‐5a^S^. When the latter domain was fused to the Sw‐5b LRR domain, NS_M_‐dependent HR triggering was regained, but not in the presence of its own Sw‐5a^S^ LRR domain. Expression analysis *in planta* revealed a nucleocytoplasmic localization pattern of Sw‐5b, in which the SD‐CC domain seems to be required for nuclear translocation. Although the Sw‐5 N‐terminal CC domain, in contrast with *Rx*, contains an additional SD, most findings from this study support a conserved role of domains within NB‐LRR (NLR) proteins against plant viruses.

## Introduction

*Tomato spotted wilt virus* (TSWV) is a member of the genus *Tospovirus* (family *Bunyaviridae*) and is responsible for significant damage to crop production around the world (Pappu *et al*., 2009). From this perspective, TSWV has been ranked as the second most important plant virus (Scholthof *et al*., 2011). In this context, two natural single dominant resistance sources have been found and are being used for commercial resistance breeding against TSWV: *Tsw* and *Sw‐5*. The first was identified in *Capsicum chinense* Jacq. ‘PI’ and has been commercially introgressed into sweet pepper (*Capsicum annuum*) cultivars (Boiteux, 1995; Jahn *et al*., 2000). The second, *Sw‐5*, was originally identified in *Solanum peruvianum* and presents a gene cluster in which five paralogues, named *Sw‐5a* to *Sw*‐*5e*, have been described (Brommonschenkel *et al*., 2000; Spassova *et al*., 2001). From this cluster, only the copy ‘*b*’ has been shown to be functionally active and to confer resistance not only to TSWV, but also to the more distantly related tospovirus species *Tomato chlorotic spot virus* (TCSV), *Groundnut ringspot virus* (GRSV) and *Impatiens necrotic spot virus* (INSV) (Hallwass *et al*., 2014; Spassova *et al*., 2001). This broad resistance spectrum is rather unique for a dominant resistance (*R*) gene and renders *Sw‐5* an interesting target for resistance breeding against tospoviruses.

Viruses have an intracellular infection cycle and, as such, are only perceived by (intracellular) R proteins. A single viral protein is usually the avirulence determinant (Avr) responsible for triggering *R* gene‐mediated resistance (Hallwass *et al*., 2014; de Ronde *et al*., 2013; Takahashi *et al*., 2001; Wen *et al*., 2013). For Sw‐5b‐mediated resistance, the non‐structural cell‐to‐cell movement protein (NS_M_) of TSWV has been identified as Avr (Hallwass *et al*., 2014, Peiro *et al*., 2014). The most commonly accepted model to explain *R* gene‐mediated resistance involves indirect sensing of Avr as presented by the ‘guard’ and ‘decoy’ hypotheses. In the first hypothesis, the *R* gene product guards a specific host protein (called the guardee) and is able to sense changes in this protein on interaction with the Avr, which triggers the resistance response (Dangl and Jones, 2001). The decoy hypothesis mainly questions the function of the ‘guardees’, seeing them as ‘decoys’ of effectors without any other roles in the cell, just evolving for this purpose (van der Hoorn and Kamoun, 2008). Although most resistance responses still remain an enigma, the activation of *R* genes (proteins) is usually accompanied by an additional triggering of a hypersensitive cell death response (HR), a programmed cell death mechanism that leads to the formation of necrotic spots and prevents further spread of the pathogen from the primary infection site (Soosaar *et al*., 2005). Dominant *R* genes can be grouped into two classes, i.e. those encoding nucleotide‐binding leucine‐rich repeat (NLR) domains, presenting the major class, and all others (de Ronde *et al*., 2014a).

The NLR proteins share structural homology with innate immunity sensors from animal cell systems (van der Biezen and Jones, 1998; Medzhitov, 2001) and commonly have, at their N‐terminal end, either a Toll and interleukin‐1 receptor (TIR) domain or a coiled‐coil (CC) domain (Meyers *et al*., 2003). Thus, these NLR proteins are also classified and referred to as TNL or CNL proteins, respectively. All *Sw‐5* genes encode CNL proteins (Meyers *et al*., 2003; Spassova *et al*., 2001), in which the N‐terminal CC domain is followed by an NB‐ARC [nucleotide‐binding adaptor shared by Apaf‐1 (from humans), R proteins and CED‐4 (from nematodes)] domain, and a leucine‐rich repeat (LRR) domain at the C‐terminus (Leipe *et al*., 2004). Nevertheless, in comparison with other CNL proteins, the CC domain of Sw‐5 proteins is extended with another subdomain, the so‐called SD (Solanaceae domain), as found earlier and reported in other Solanaceae NLR proteins (Mucyn *et al*., 2006). Although functions for these major domains have been explored for some CNL proteins and mechanistic models have been proposed (Takken and Goverse, 2012), many differences are seen that make it difficult to present a single activation model that applies to all proteins.

In this study, the genetic basis for resistance or susceptibility to TSWV was investigated with three homologues of *Sw‐5*, i.e. the known functional resistance gene copy (*Sw‐5b*), its highest conserved paralogue from *S. peruvianum* (*Sw‐5a*) and their highest conserved orthologue from susceptible *S. lycopersicum* (*Sw‐5a^S^*). It is shown that the Sw‐5b protein is able to trigger HR in an NS_M_‐dependent and ‐independent manner (auto‐activation), whereas Sw‐5a is only able to do this independently and Sw‐5a^S^ does not trigger HR under any condition. The SD‐CC, NB‐ARC and LRR domains were each further investigated for these abilities, and the results are discussed in the light of the ability of *Sw*−5 proteins to confer resistance against tospoviruses.

## Results

### High expression levels induce Sw‐5b auto‐activation

So far, only Sw‐5b has been shown to halt tospovirus infections, although its paralogue Sw‐5a, which shares almost 95.1% amino acid sequence identity, does not. To study the genetic basis for resistance or susceptibility to TSWV, Sw‐5a and Sw‐5b were further analysed and dissected into their respective SD‐CC, NB‐ARC and LRR domains for their role in Sw‐5‐mediated HR. Using a specific primer set for the amplification of *Sw‐5a/b*, an additional homologue was amplified from susceptible tomato plants, from here onwards referred to as *Sw‐5a^S^*. This homologue is seen in the Heinz 1706 tomato genome sequence available at the National Center for Biotechnology Information (NCBI) (Sato *et al*., 2012). Although there are other *Sw‐5* paralogues in the Heinz 1706 genome (Andolfo *et al*., 2014), Sw‐5a^S^ is the closest homologous protein to Sw5‐a/b, with a slightly higher amino acid identity to Sw‐5a (94.7%) than to Sw‐5b (94.2%).

**Figure 1:**
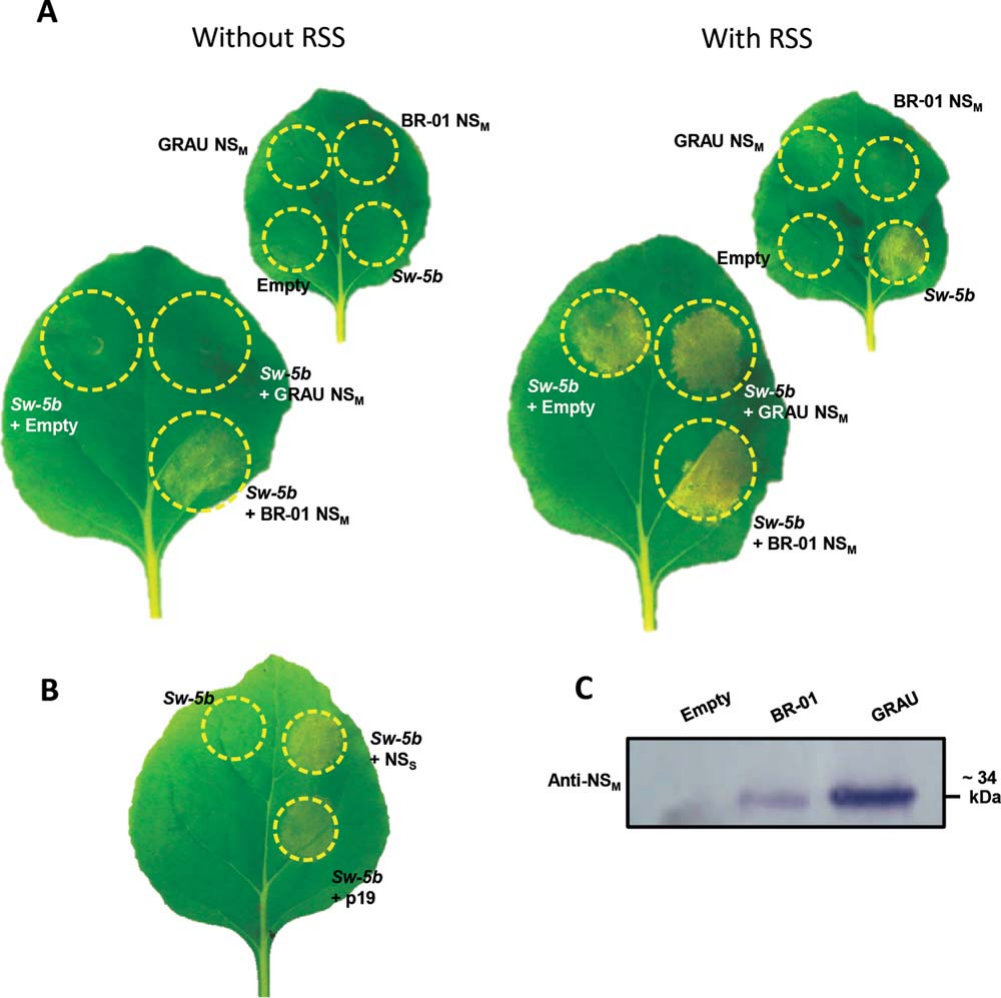
NS_M_‐dependent and NS_M_‐independent induction of Sw‐5b‐mediated hypersensitive cell death response (HR). (A) The two leaves of *Nicotiana benthamiana* in higher magnification were agroinfiltrated with constructs of BR‐01‐ or GRAU‐NS_M_ and *Sw‐5b*, cloned into pK2GW7 (left leaf) or pEAQ‐HT [right leaf; pEAQ‐HT additionally contains the RNA silencing suppressor (RSS) p19 gene in its backbone]. As negative controls, all constructs were singly infiltrated (lower magnification leaves) with Sw‐5b expressed from pK2GW7 (left) and pEAQ‐HT (right). (B) Co‐infiltration of agrobacteria harbouring the *Sw‐5b* gene (cloned into pK2GW7) and NS_S_ or p19 (cloned into pK2GW7 or pBIN). (C) Western immunoblot analysis of NS_M_ proteins expressed from pK2GW7. The expression of NS_M_ constructs from pEAQ‐HT has been reported and described previously (Hallwass *et al*., 2014). Photographs were taken at 4 days post‐agroinfiltration (dpa).

To further dissect the genetic basis for resistance or susceptibility for different Sw‐5 proteins, an alternative to the time‐consuming use of transgenic *Nicotiana benthamiana* lines (Hallwass *et al*., 2014) had to be developed. To this end, and as a first step, the functional resistance gene copy *Sw‐5b* was cloned into two different binary vectors, i.e. pK2GW7 and pEAQ‐HT (Karimi *et al*., 2002; Peyret and Lomonossoff, 2013), and tested in transient settings in wild‐type *N. benthamiana*. Although the expression level of genes cloned into pK2GW7 relies on the 35S promoter and, in general, is sufficient for most research purposes, genes obtained from pEAQ‐HT generally show higher expression as a result of enhancement by flanking *Cowpea mosaic virus* RNA2 leader/trailer sequences and suppression of transgene silencing by the *Tombusvirus*‐P19 RNA silencing suppressor (RSS) gene in the vector backbone (Peyret and Lomonossoff, 2013). When constructs of *Sw‐5b* were co‐agroinfiltrated with TSWV NS_M_ constructs from either the resistance‐breaking (RB) GRAU isolate (which does not trigger *Sw‐5b*/HR) or the resistance‐inducing (RI) BR‐01 isolate (Hallwass *et al*., 2014), a specific Avr‐triggered HR was only observed with NS_M_ (RI) during co‐expression with Sw‐5b constructs expressed from pK2GW7 (Fig. 1A). However, when expressed from pEAQ‐HT‐based constructs, Sw‐5b was able to trigger HR in the absence of the NS_M_ (RI) protein (Fig. 1A). In the control samples, no cell death response was triggered by the ‘empty’ pEAQ‐HT vector, that still expressed p19 from its vector backbone, nor when this vector was infiltrated into *Sw‐5b*‐transgenic *N. benthamiana* or in *Sw‐5*‐containing tomato lines (Hallwass *et al*., 2014), ruling out the possibility that p19 triggered an HR. These data suggest that the auto‐activity of Sw‐5b is probably induced by higher expression levels obtained from pEAQ‐HT, in which co‐expression with the RSS p19 prevents silencing and stabilizes higher expression levels, as described by Peyret and Lomonossoff (2013), and previously observed during our own studies on dysfunctional TSWV NS RSS mutants (de Ronde *et al*., 2014b). High protein accumulation leading to auto‐activity in the presence of p19 has been reported previously for the CNL protein NRC1 (Gabriels *et al*., 2007). To further support this idea, a similar experiment was performed in which Sw‐5b was expressed from pK2GW7 in the absence or additional presence of a binary vector from which the RSS protein P19 or NS_S_ was expressed. Although, in the absence of RSS proteins, no HR was triggered, auto‐activation was observed in the presence of either p19 or NS_S_ (Fig. 1B). Expression of the agroinfiltrated TSWV NS_M_ gene constructs from BR‐01 and GRAU isolates was verified by western immunoblot analysis (Fig. 1C).

### Sw‐5a protein also triggers cell death by auto‐activation, but fails to recognize NS_M_ as Avr, whereas Sw‐5a^S^ lacks both functions

Having established a transient expression system that discriminates between NS_M_‐dependent and NS_M_‐independent HR triggering (by co‐infiltration with RSS), we set out to separately analyse effector recognition and HR activation of other Sw‐5 homologues. Initial tests using the functional *Sw‐5b* resistance gene copy demonstrated that green fluorescent protein (GFP) and His‐Tag fusions at both N‐ and C‐terminal ends did not affect its HR triggering activity. As a result of the lack of antibodies against the Sw‐5 protein, and to be able to verify protein expression, all Sw‐5 proteins and their derived constructs used from this point onwards were fused at their N‐terminus either to GFP (pK7WGF2) or to a His‐Tag (pEAQ‐HT‐DEST2).

**Figure 2:**
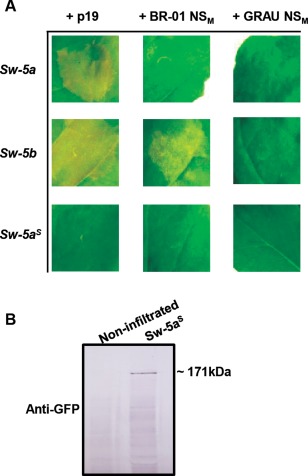
NS_M_‐dependent and NS_M_‐independent hypersensitive cell death response (HR) induction by Sw‐5 orthologues. (A) Analysis of HR induction by Sw‐5a and Sw‐5a^S^ was performed in a similar manner to the experiment described in Fig. 1 and using the functional Sw‐5b as a positive control. In the first column of (square) images, *Nicotiana benthamiana* leaves were co‐infiltrated with agrobacteria harbouring *Sw‐5a*, *Sw‐5b* or *Sw‐5a^S^* (cloned into pK7WGF2) and p19 for cell death triggering by auto‐activation. In the other columns, images are shown from leaf areas co‐infiltrated with agrobacteria harbouring the same *Sw‐5* constructs and BR‐01‐NS_M_ (second column) or GRAU‐NS_M_ (third column), all constructs cloned in pK2GW7. Images were taken at 4 days post‐agroinfiltration (dpa). (B) Western immunoblot detection of Sw‐5a^S^ in leaves from (A) (first column) co‐infiltrated with pK7WGF2 constructs of Sw‐5a^S^ and p19. Western blot was screened with anti‐green fluorescent protein (anti‐GFP) as primary antibody.

Sw‐5a and Sw‐5a^S^ proteins were tested with regard to their ability to trigger HR in an NS_M_‐dependent and NS_M_‐independent manner. Although Sw‐5a, like the (GFP‐fused) Sw‐5b, auto‐activated when co‐expressed with p19, in contrast, it failed to trigger HR when co‐expressed with BR‐01 NS_M_ (Fig. 2A). Sw‐5a^S^, on the other hand, was unable to trigger HR at all (Fig. 2A). Together, these data indicate that Sw‐5a still maintains the ability to trigger a downstream pathway leading to HR, but fails to (in)directly sense the viral Avr NS_M_, whereas Sw‐5a^S^ appears to be non‐functional in both features. As a result of the early onset of cell death triggering (in the presence of p19), we were unable to visualize the presence of full‐length Sw‐5a and Sw‐5b proteins on a western immunoblot in extracts from agroinfiltrated *N. benthamiana* leaves. However, their auto‐activation (Sw‐5a and Sw‐5b) and NS_M_‐dependent HR (Sw‐5b), in addition to the dissection of these proteins as described in the following sections, were in clear support of their expression. The Sw‐5a^S^ protein, in contrast, was well detected from leaves co‐infiltrated with pK7WGF2 constructs of Sw‐5a^S^ and p19 (Fig. 2B), and did not reveal any auto‐activation.

### The Sw‐5b NB‐ARC domain is sufficient to trigger HR

**Figure 3:**
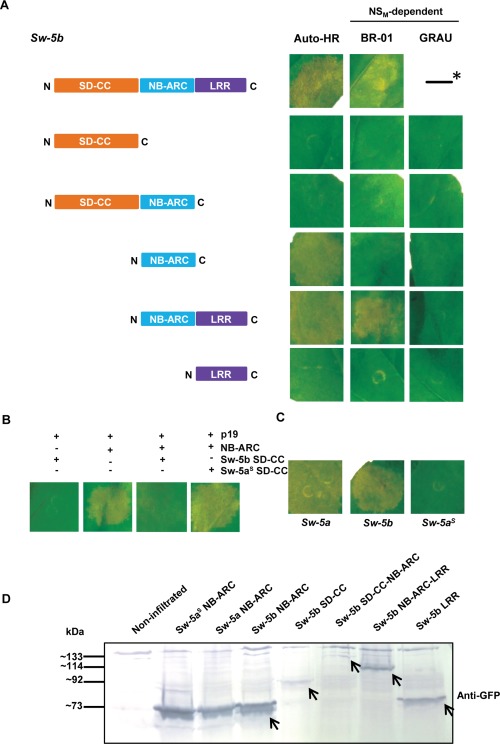
NS_M_‐dependent and NS_M_‐independent hypersensitive cell death response (HR) induction by domains from Sw‐5 orthologues. (A) Co‐infiltration of agrobacteria harbouring *Sw‐5b* gene‐based derivatives (cloned into pK7WGF2) as depicted, with p19 (first column), BR‐01 NS_M_ (second column) and GRAU NS_M_ (third column). All constructs were always infiltrated into the same leaf per assay. Photographs were taken at 4 days post‐agroinfiltration (dpa). *As a positive control on (auto)HR triggering, the *Sw‐5b* full‐length gene was co‐infiltrated with p19 (first column; auto‐HR) or BR‐01‐NS_M_ (second column; NS_M_‐dependent). (B) Co‐infiltration of *Nicotiana benthamiana* with agrobacteria harbouring the NB‐ARC and/or SD‐CC domains as depicted, and with p19 (from pBIN) for cell death triggering by auto‐activation. Leaves were monitored for HR at 4 dpa. Independent of the combination, each construct was infiltrated at the same agrobacterium optical density (OD). (C) Co‐infiltration of agrobacteria harbouring the NB‐ARC domains from *Sw‐5a*, *Sw‐5b* or *Sw‐5a^S^* and p19 for auto‐HR triggering; photographs were taken at 4 dpa. (D) Western blot of the Sw‐5b‐truncated proteins expressed via pK7WGF2‐based constructs. Anti‐green fluorescent protein (anti‐GFP) was used as primary antibody. ARC, adaptor shared by Apaf‐1 (from humans), R proteins and CED‐4 (from nematodes); CC, coiled coil; LRR, leucine‐rich repeat; NB, nucleotide binding. SD, Solanaceae domain.

In order to assign the ability to trigger HR or sense Avr to any of the SD‐CC, NB‐ARC or LRR domains, truncated constructs were made that covered each of these individual domains or combinations (SD‐CC‐NB‐ARC, NB‐ARC‐LRR) from Sw‐5b (Fig. 3A). All Sw‐5b‐based proteins were expressed in the presence of p19 or NS_M_ to test for auto‐activation and Avr recognition, respectively (Fig. 3A). In addition to the full‐length Sw‐5b protein (positive control), cell death by auto‐activation was also triggered by the NB‐ARC and NB‐ARC‐LRR domains, but not with SD‐CC‐NB‐ARC (Fig. 3A). The absence of any auto‐activation by SD‐CC‐NB‐ARC was not simply a result of low expression levels, as co‐expression of NB‐ARC with the SD‐CC domain from two separate constructs also did not show auto‐activation (Fig. 3B). These findings indicate that the NB‐ARC domain is sufficient for HR triggering, a process that can be suppressed by SD‐CC *in cis* as well as *in trans*. Interestingly, when the SD‐CC domain from Sw‐5a^S^ was co‐expressed with the Sw‐5b NB‐ARC domain, no HR suppression was observed. On expression of all Sw‐5b truncations and individual domain constructs in the presence of BR‐01 NS_M_, Avr‐dependent HR triggering was only observed with NB‐ARC‐LRR (Fig. 3A), suggesting that the LRR domain is required for Avr recognition. In addition, expression of the NB‐ARC domain from Sw‐5a also triggered cell death by auto‐activation, whereas that from Sw‐5a^S^ did not (Fig. 3C), in agreement with the results obtained when using their full‐length genes (Fig. 2). All Sw‐5b‐derived gene constructs were confirmed for protein expression (Fig. 3D), in which only the constructs containing an SD‐CC domain revealed lower expression levels.

### The non‐functional Sw‐5a^S^ NB‐ARC domain regains the ability to trigger cell death by auto‐activation on a single amino acid reversion

**Figure 4:**
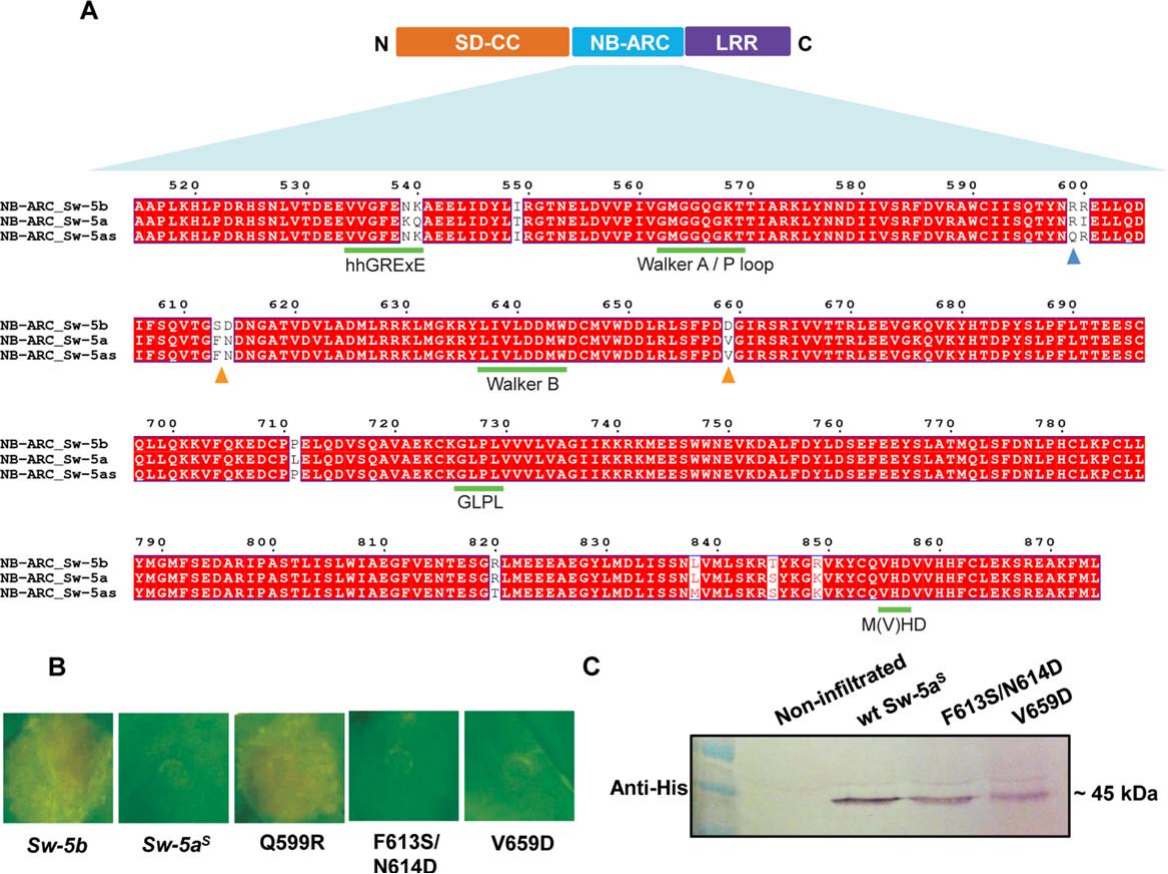
Analyses of mutant Sw‐5a^S^ NB‐ARC domains in cell death triggering. (A) Multiple sequence alignment of NB‐ARC domains from different Sw‐5 homologues. The blue triangle indicates a mismatch of Sw‐5a^S^ with both Sw‐5a and Sw‐5b, and orange triangles indicate mismatches only seen with Sw‐5b. Green bars indicate conserved motifs previously identified for NB‐LRRs (NLRs) (Van Ooijen *et al*., 2008) (B) *Nicotiana benthamiana* leaves agroinfiltrated with pEAQ‐DEST2 constructs (which also contain p19) of NB‐ARC domains from wild‐type Sw‐5b, Sw‐5a^S^ and mutants Q599R, F613S/N614D and V659D prepared from the Sw‐5a^S^ NB‐ARC domain. Photographs were taken at 4 days post‐agroinfiltration (dpa). (C) Western immunoblot analysis to verify the expression of NB‐ARC constructs that negatively tested for HR triggering in (B). Anti‐histidine (anti‐His) was used as primary antibody. ARC, adaptor shared by Apaf‐1 (from humans), R proteins and CED‐4 (from nematodes); CC, coiled coil; LRR, leucine‐rich repeat; NB, nucleotide binding. SD, Solanaceae domain.

**Figure 5:**
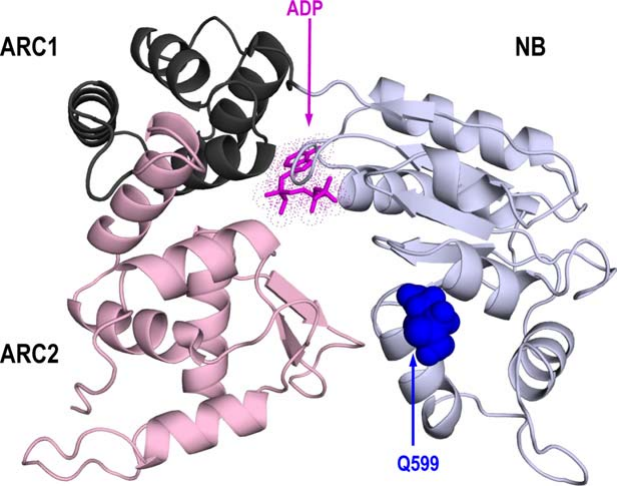
Three‐dimensional structural model of the Sw‐5a^S^ NB‐ARC domain. The location of Q599 is indicated in a structural model of the Sw5 NB site in its closed ADP state. The NB region is shown in blue–white, ARC1 in dark grey and ARC2 in light pink; ADP is shown in magenta. ADP, adenosine diphosphate; ARC, adaptor shared by Apaf‐1 (from humans), R proteins and CED‐4 (from nematodes); NB, nucleotide binding.

As the NB‐ARC domain was sufficient to trigger HR, a multiple amino acid sequence alignment was performed from the NB‐ARC domains of Sw‐5 homologues to identify potential amino acid residues that could be responsible for the lack of auto‐activity of Sw‐5a^S^ from susceptible tomato. Based on the alignment, three putative amino acid residues were identified in the NB‐ARC domain sequence of Sw‐5a^S^ that clearly differed from those of the others (Fig. 4A). These were reversed one by one into the corresponding amino acid residue present in the Sw‐5b NB‐ARC domain (Fig. 4A). Binary constructs (pEAQ‐HT‐DEST2) of the three revertants were infiltrated into *N. benthamiana*, next to positive (Sw‐5b NB‐ARC) and negative (Sw‐5a^S^ NB‐ARC) controls, and tested for auto‐activation. Interestingly, a gain of function was observed with the mutant Q599R (Fig. 4B), but not with the others. The absence of auto‐activation from Sw‐5a^S^ NB‐ARC, Sw‐5a^S^ F613S/N614D and Sw‐5a^S^ V659D was not a result of the absence of protein expression, as the proteins were well detectable by their His‐tag on western immunoblots (Fig. 4C). In an attempt to explain the gain of auto‐activity on the Q599R mutation, structural folding was predicted for the NB‐ARC domain as described in Slootweg *et al*. (2013) and starting from the crystal structure of APAF‐1 (see Experimental procedures). This folding interestingly revealed that the Q599R mutation was localized on the edge of the NB subdomain, and clearly outside of the ATP/ADP‐binding pocket (Fig. 5).

### The LRR domain from Sw‐5b fused to the Q599R NB‐ARC revertant from Sw‐5a^S^ activates NS_M_‐dependent HR

**Figure 6:**
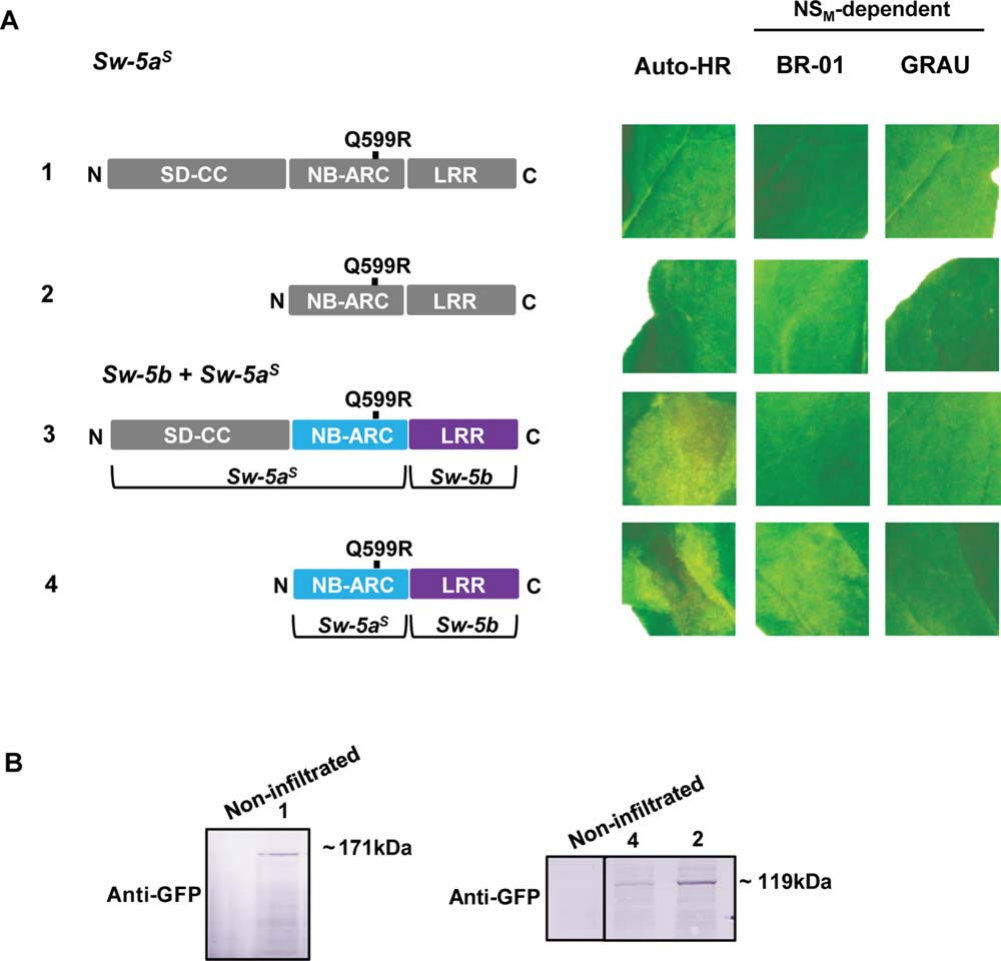
NS_M_‐dependent and NS_M_‐independent hypersensitive cell death response (HR) induction by Sw‐5a^S^ chimera. (A) Co‐agroinfiltration of (mutant) *Sw‐5a^S^* gene‐based constructs (cloned into pK7WGF2) with p19 (first column; auto‐activity), BR‐01‐NS_M_ (second column; NS_M_‐dependent) or GRAU‐NS_M_ (third column; NS_M_‐dependent) in *Nicotiana benthamiana* leaves. Photographs were taken at 4 days post‐agroinfiltration (dpa). (B) Western immunoblot detection of (mutant/chimeric) Sw‐5a^S^ proteins from (A). Anti‐green fluorescent protein (anti‐GFP) was used as primary antibody. ARC, adaptor shared by Apaf‐1 (from humans), R proteins and CED‐4 (from nematodes); CC, coiled coil; LRR, leucine‐rich repeat; NB, nucleotide binding. SD, Solanaceae domain.

Having identified Q599R as an important amino acid change within the Sw‐5a^S^ NB‐ARC domain to restore its ability to induce HR, this mutation was next introduced into the full‐length Sw‐5a^S^ (Fig. 6). However, when this construct was expressed in *N. benthamiana*, no auto‐ (from pEAQ‐HT) or NS_M_‐dependent (from pK7WGF2) HR was observed (Fig. 6A). In the light of the differences in the LRR domains described previously, the LRR domain from the full‐length Sw‐5a^S^ was next removed and replaced by the LRR domain from Sw‐5b. Considering that this chimeric Sw‐5 contained an NB‐ARC domain restored in its function with regard to cell death and an LRR domain functional in Avr recognition, it was surprising that only cell death by auto‐activation was visualized on infiltration of the construct into *N. benthamiana* (Fig. 6A). To determine whether this was caused by the SD‐CC domain from Sw‐5a^S^, this domain was removed from the mutated (Q599R) full‐length Sw‐5a^S^ and from the chimeric Sw‐5a^S^/Sw‐5b protein (Fig. 6A). Interestingly, auto‐ and NS_M_‐dependent HR was observed for the chimeric NB‐ARC(Q599R)‐LRR construct, indicating an interference of the Sw‐5a^S^ SD‐CC domain during effector recognition. The constructs were verified for expression by western immunoblot detection (Fig. 6B).

### The SD‐CC domain of Sw‐5b is required for nuclear translocation

**Figure 7:**
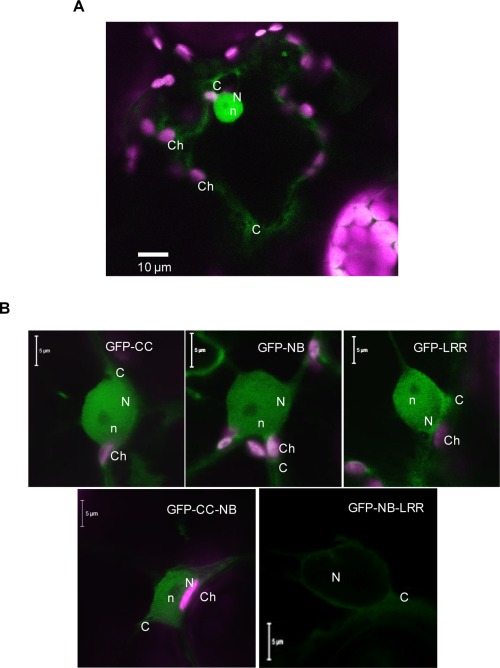
Subcellular localization of full‐length and truncated Sw‐5b proteins fused to green fluorescent protein (GFP) in *Nicotiana benthamiana* leaves. (A) Transient expression of the full‐length Sw‐5b protein. (B) Expression of truncated Sw‐5b proteins. Confocal images were captured at 4 days post‐agroinfiltration (dpa). C, cytoplasm; Ch, chloroplast; n, nucleolus; N, nucleus.

Several studies on *R* gene products have revealed a nucleocytoplasmic distribution, and this has been explained to be required for the triggering of resistance and HR (for a current overview, see table 1 in Wang and Balint‐Kurti, 2015). To analyse the cellular distribution of Sw‐5b and the role of different domains in this, Sw‐5b‐GFP fusion constructs and derivatives were transiently expressed in leaves of *N. benthamiana* and analysed by confocal scanning microscopy. Although SD‐CC domain‐containing proteins were normally harder to detect on a western immunoblot (Fig. 3D), they could be well detected in the cell by fluorescence microscopy, like most other constructs (Fig. 7). The results also showed that GFP‐Sw‐5b localized in both the nucleus and cytoplasm (Fig. 7A), and this was similarly observed for the individual domains and GFP‐SD‐CC‐NB‐ARC (Fig. 7B). In contrast, GFP‐NB‐ARC‐LRR was only observed in the cytoplasm and seemed absent from the nucleus, which indicated that SD‐CC appears to promote nuclear import. Furthermore, the results supported the earlier indications that the Sw‐5b protein, which could not be detected on a western immunoblot in extracts from agroinfiltrated *N. benthamiana* leaves during auto‐activation or NS_M_‐dependent triggering of HR, was expressed.

## Discussion

In this study, the functional Sw‐5b resistance protein and its highly conserved paralogue (Sw‐5a) from *S. peruvianum* and orthologue (Sw‐5a^S^) from susceptible *S. lycopersicum* were comparatively analysed to identify the role of the SD‐CC, NB‐ARC and LRR domains in HR induction and NS_M_ recognition as Avr. In this way, we aimed to understand the genetic basis for the inability to confer resistance to TSWV by Sw‐5a and Sw‐5a^S^. To circumvent the time‐consuming generation of transgenic plants, a transient system to express Sw‐5 homologues, their domains and chimeric versions was established. At higher levels of protein expression (as a result of the additional presence of the RSS p19), the functional Sw‐5b was able to auto‐activate in the absence of the NS_M_ Avr determinant, whereas, at lower levels, HR triggering required NS_M_ recognition. Using these assays, cell death triggering and NS_M_ recognition were investigated for the aforementioned three Sw‐5 proteins. Sw‐5a was still able to trigger cell death, but failed to do so in an NS_M_‐dependent fashion. This observation explains why *Sw‐5a*‐transformed tobacco plants still exhibit a TSWV‐susceptible phenotype (Spassova *et al*., 2001). *Sw‐5a^S^*, however, failed to trigger HR at all.

The observation that high levels of Sw‐5b accumulation in cells leads to an auto‐immune scenario has been reported previously for other R proteins, such as RPS2, RPS4 and Pto (Day *et al*., 2005; Tang *et al*., 1999; Zhang *et al*., 2004), but cases have also been reported in which increased expression levels, instead of resulting in auto‐activation, lead to an increased (extreme) resistance response (Sato *et al*., 2014). The screening of *R* genes by HR remains controversial because disease resistance and HR triggering have been demonstrated to be two events that can be uncoupled for *Arabidopsis* RPS4, potato Rx and barley Rrs1 (Coll *et al*., 2011; Heidrich *et al*., 2011; de Ronde *et al*., 2014a). For the *Sw‐5b* gene and most other dominant resistance genes, however, HR is commonly observed on resistance activation, and therefore presents a reliable visual indicator of protein activation (Hallwass *et al*., 2014; de Ronde *et al*., 2013).

Analysis of the domains from functional Sw‐5b demonstrated that NS_M_ recognition mapped, as expected, to the LRR domain, whereas the induction of HR required the NB‐ARC domain. Although the SD‐CC domain was not relevant for these functions, the *in cis* or *in trans* presence of SD‐CC next to NB‐ARC lacking LRR suppressed HR induction. From the other two homologues, Sw‐5a contained a functional NB‐ARC domain, but failed to recognize NS_M_ by its LRR domain. On the other hand, all three domains of Sw‐5a^S^ from susceptible *S. lycopersicum* appeared to be hampered or changed in their ability to trigger cell death (NB‐ARC) or NS_M_‐dependent HR (LRR). However, a single point mutation in NB‐ARC was sufficient for a gain of function to trigger cell death by auto‐activation, which was not entirely surprising considering the very high protein sequence identity (95%) shared with Sw‐5b. After alignment of the available *Sw‐5* protein sequences from *S. peruvianum* and *S. lycopersicum* Heinz (Fig. S2 and Table S2, see Supporting Information), Sw‐5a^S^ is the only sequence that presents the point mutation R599Q related to loss of function. Whether the NB‐ARC domains from other (non‐tested) Sw‐5 homologues lacking this mutation are able to trigger cell death by auto‐activation still remains to be investigated, considering that more polymorphisms are seen among these. The NB‐ARC domains have been hypothesized to work as a switch, with active and inactive states according to ATP and ADP binding, respectively (Qi *et al*., 2013). However, a structural folding prediction of Sw‐5a^S^ NB‐ARC based on the Rx NB‐ARC structural template revealed that the R599Q mutation is not located near the ADP/ATP‐binding site. This suggests that ATP binding does not seem to be a cause of the lack of auto‐activity, but rather a change in the structure–function relationship may lead to a loss of HR, e.g. by affecting DNA binding, as described by Fenyk *et al*. (2015).

The sole requirement of the NB‐ARC domain for HR triggering has also been reported previously for potato Rx (Rairdan *et al*., 2008). Nevertheless, CC and TIR domains have also been associated with downstream signalling that leads to HR triggering, as observed for barley MLA10, maize Rp1‐D21 and flax L6 (Bernoux *et al*., 2011; Maekawa *et al*., 2011; Wang *et al*., 2015), and CC‐NB‐ARC for Arabidopsis RPS5 (Ade *et al*., 2007). For Sw‐5b, the SD‐CC domain suppressed HR, an observation that has been made previously with the tomato Mi‐1.2 resistance gene and specifically mapped to SD, the first subdomain localized in the N‐terminus of this protein (Lukasik‐Shreepaathy *et al*., 2012). A closer look at the Sw‐5 proteins also revealed the presence of a putative SD domain (Fig. S1, see Supporting Information), but, in this study, the entire N‐terminal region of Sw‐5 was denominated as a single domain (SD‐CC), as coiled‐coil secondary structures are predicted to span the entire amino acid sequence up to the start of the NB‐ARC domain. As mentioned previously by Mucyn *et al*. (2006), this extended N‐terminal region presents a weak homology among solanaceous R proteins and has been arbitrarily named the SD and N‐terminal domain. Whether the HR suppression indeed involves either the SD domain or the N‐terminal domain requires a further dissection of the Sw‐5b SD‐CC domain. The presence of several single nucleotide polymorphisms (SNPs) in the SD‐CC domain from Sw‐5a^S^ might also explain why this domain was unable to suppress HR during co‐expression with Sw‐5b NB‐ARC. From the various Sw‐5b truncations or individual domains tested, GFP‐NB‐ARC‐LRR localized exclusively in the cytoplasm, whereas all other fusion proteins showed a nucleocytoplasmic distribution similar to the full‐length Sw‐5b protein. These data seem to suggest an important role for the SD‐CC domain in nuclear translocation. The fusion proteins GFP‐NB‐ARC and GFP‐LRR, containing single domains, were also seen in the nucleus. However, their localization might be a result of passive diffusion into the nucleus, as indicated by earlier observations in which even proteins larger than 60 kDa can diffuse through the nuclear pore complex (Wang and Brattain, 2007). Furthermore, we also cannot rule out the possibility that this signal is caused by (‘free’) GFP originating from the degradation products of the full‐length fusion proteins, although no smaller products were observed during the western immunoblot detection of Sw‐5 GFP fusion proteins. In general, the LRR domain seems to be most conserved in its role of activating R proteins, but other domains have also been reported to interact with effectors, e.g. the heavy metal‐associated (HMA) domain of the rice Pik protein (Maqbool *et al*., 2015). Based on these differences, to date, it is difficult to postulate one mechanistic model to explain the activation of all NLR proteins. Apart from potato Rx and *Tobacco mosaic virus* (TMV) resistance protein N (Mestre and Baulcombe, 2006; Whitham *et al*., 1994), no other *NLR* genes against viruses have been studied in extensive detail. The results shown here for Sw‐5 proteins are more or less in agreement with what has been found to date for Rx and a few other resistance genes against viruses, and provide the first evidence as to why only Sw‐5b is functional against tospoviruses. Although the mechanism of Sw‐5b resistance has not yet been elucidated, the information from this study will be helpful for tomato breeding and contribute to the screening of potential resistant cultivars. Furthermore, considering that paralogues may trigger resistance to different types of pathogen, as well exemplified by potato Rx and Gpa2, which provide resistance to *Potato virus X* (PVX) and the nematode *Globodera pallida* (van der Vossen *et al*., 2000), respectively, this possibility should not be ruled out for other Sw‐5 proteins.

The assembly of artificial R proteins seems to be very tricky as a very stable combination of domains is required. On this point, earlier studies involving domain swaps have resulted in the inactivation of other NLR proteins as well (Rairdan and Moffett, 2006; Sun *et al*., 2001). In this study, a gain of function was observed by the introduction of the Q599R mutation in the Sw‐5a^S^ NB‐ARC alone, but not in the full‐length protein. Deletion of its own SD‐CC domain, for which the counterpart of Sw‐5b suppresses auto‐activity, was not sufficient to retrieve the auto‐activity in the reverted full‐length Sw‐5a^S^ protein. Only when its LRR domain was replaced by that from Sw‐5b, and the SD‐CC domain was deleted, was the ability to trigger both auto‐ and NS_M_‐dependent HR regained. Altogether, our data support the idea that changing only a few amino acid residues might change an apparently non‐functional R protein into a functional one. With the recent availability of site‐specific DNA editing tools, such as TALENs (transcription activator‐like effector nucleases) or CRISPR (clustered regularly interspaced short palindromic repeats) (Gaj *et al*., 2013), mapped gain‐of‐function mutations could be introduced into the genome of susceptible plants.

## Experimental Procedures

### Plants

The seeds of the tomato near‐isogenic isolines ‘Santa Clara’ and ‘CNPH LAM 147’ (Hallwass *et al*., 2014) were kindly supplied by Dr Leonardo Boiteux (EMBRAPA Vegetables, Brasilia, Brazil). Both *N. benthamiana* and tomato plants were kept under glasshouse conditions (24 ºC, 16 h light/8 h dark per day).

### Cloning into binary vectors

DNAZOL® reagent (Invitrogen, Carlsbad, CA, USA) was used for total DNA extraction of the tomato near‐isogenic lines, which was then employed as template for the amplification of the *Sw‐5* genes (*Sw‐5a*, *Sw‐5a^S^*, *Sw‐5b*) via polymerase chain reaction (PCR) using Phusion Taq DNA polymerase (Thermo Scientific, Waltham, MA, USA). The PCR products were recombined into pDONR207 entry vectors by BP Clonase Enzyme Mix (Invitrogen). Truncated gene versions were PCR amplified from their full‐length copies, cloned in pDONR207 vectors, and either ligated in pENTR11 (*Nco*I and *Xho*I restriction sites) or recombined again in pDONR207, as detailed in Table S1 (see Supporting Information). The pDONR207 constructs harbouring NS_M_ from the RI BR‐01 or RB GRAU strains were prepared previously (Hallwass *et al*., 2014). All entry vectors were recombined with one or more of the following destination vectors by LR Clonase Enzyme Mix (Invitrogen): pEAQ‐DEST1, pEAQ‐DEST2, pK2GW7, pK7WGF2 (Karimi *et al*., 2002; Peyret and Lomonossoff, 2013). A list of all primer sequences, entry and destination vectors of all constructs used in this study is shown in Table S1. All procedures followed the manufacturer's instructions and Green and Sambrook (2012).

### Mutagenesis PCR and cloning

The pDONR207 vectors harbouring the full or truncated gene versions were used as template for overlapping PCR, employing primers to insert the desired mutations. After amplification with Phusion DNA polymerase, 1 µL of *Dpn*I restriction enzyme (NEB, Ipswich, MA, USA) was added for template DNA degradation. To facilitate rapid and easy screening of mutant constructs, restriction sites were introduced or removed via silent mutations. The chimeric *Sw‐5* gene was made by fusion PCR, first amplifying the sequences separately and next fusing them during another PCR. Fused PCR products were then cloned into pDONR207. All constructs from the entry vectors were recombined with pEAQ‐DEST2 or pK7 as listed in Table S1.

### Agroinfiltration, protein extraction and western blot analysis

The agroinfiltration assays were performed following the protocol of Bucher *et al*. (2003). Thus, *Agrobacterium tumefaciens* COR308 containing single constructs was grown overnight at 28°C in LB3 medium with 2 µg/mL tetracycline and either 250 µg/mL spectinomycin for pK2 and pK7 vectors or 100 µg/mL kanamycin for pEAQ‐HT vectors. Then, 600 µL of LB3 inoculum was added to 3 mL of induction medium and, once again, grown overnight. After harvesting and resuspending the bacteria with MS‐MES buffer, *N. benthamiana* leaves were infiltrated with combinations of suspensions containing a final optical density at 600 nm (OD_600_) of 1.0 per construct. Photographs were taken at 4 days post‐agroinfiltration (dpa). Cell death assays were repeated at least three times, in which three leaves per plant and three plants per assay were infiltrated with each sample (single or combinations of agrobacteria) in the presence of positive and negative controls for HR triggering. Only samples triggering HR at least 19 times (70%) from the 27 spots agroinfiltrated were considered to be positive. No observation of HR in at least 19 of 27 spots was considered to be negative.

For protein extraction, leaves were harvested at 3 or 4 dpa for the full‐length proteins. About 100 mg of leaf material per sample were ground in liquid nitrogen and then with 250 µL of 95 ºC pre‐heated Berger buffer (Berger *et al*., 1989). Samples were incubated for 10 min at 95 ºC and centrifuged at 12 000 ***g*** for 10 min. Supernatants were transferred to new tubes and the same volume of 2 × sodium dodecylsulfate (SDS) loading buffer was added. Samples were incubated at 95 ºC for 3 min. A volume of 15 µL per sample was loaded in 10% sodium dodecylsulfate‐polyacrylamide gel electrophoresis (SDS‐PAGE) gels. The western blotting procedure was performed as described previously (Hallwass *et al*., 2014) and using anti‐His and anti‐GFP (Molecular Probes, Eugene, OR, USA) as primary antibodies. The detection was performed with alkaline phosphatase substrate.

### *In silico* analysis

For structural folding predictions, the Sw‐5 proteins were virtually divided into three main portions (Sw‐5b as reference): (01) SD‐CC, 1–514 amino acids; (02) NB‐ARC, 515–874 amino acids; and (03) LRR, 875–1246 amino acids (for sequence, see Table S2). The nucleotide and amino acid alignments were performed by ClustalW (Larkin *et al*., 2007) and Espript 3.0 (Robert and Gouet, 2014). The CC secondary structures of the Sw‐5 proteins were predicted by http://gpcr.biocomp.unibo.it/cgi/predictors/cc/pred_cchmm.cgi (Hochreiter *et al*., 2007). The NB‐ARC and LRR domains were predicted by SMART (Letunic *et al*., 2012).

### Confocal microscopy

Images of epidermal cells from *N. benthamiana* leaves were obtained using a Zeiss (Jena, Germany) LSM 510‐META 18 confocal laser scanning microscope with a ×40, 1.3‐numerical aperture, oil‐corrected objective. GFP imaging was obtained by 488‐nm excitation from an argon laser and emission was detected through a 505–570‐nm filter. Chlorophyll emission was detected through a 650‐nm filter. The parameters for image acquisition were kept the same for all constructs. Photographs were taken at 4 dpa.

### Three‐dimensional modelling of the NB‐ARC domain

Domain delineation, sequence analysis and molecular modelling of the NB‐ARC domains of Sw‐5 proteins were performed as described by Slootweg *et al*. (2013), starting from the crystal structure of APAF‐1 (PDB code 1Z6T).

## Supporting information

Additional Supporting Information may be found in the online version of this article at the publisher's website:

**Table S1.** Primers and vectors used for building and expression of the full, truncated and mutated *Sw‐5* gene versions.

**Table S2.** *Sw‐5* gene sequences from *Solanum lycopersicum* Heinz.

**Fig. S1.** Multiple sequence alignment of SD‐CC domains from Sw‐5a (AY007366), Sw‐5b (AY007366), Sw‐5a^S^ (Table S2), Mi‐1.2 (AF039682) and Rx (AJ011801). The amino acid residues shaded in green indicate a putative coiled‐coil (CC) domain in comparison with potato Rx, which lacks the so‐called Solanaceae domains (SD). The area shaded in grey covers the extended N‐terminus previously divided as the N‐terminal domain and SD for potato Prf (Mucyn *et al*., 2006) or SD1 and SD2 domains for tomato Mi‐1.2 (Lukasik‐Shreepaathy *et al*., 2012).

**Fig. S2.** Multiple sequence alignment of NB‐ARC [nucleotide‐binding adaptor shared by Apaf‐1 (from humans), R proteins and CED‐4 (from nematodes)] domains from available and full Sw‐5 protein sequences from *Solanum peruvianum* and *S. lycopersicum* Heinz. The blue bar indicates the Q599R mutation. GenBank accessions: AY007366 (Sw‐5a and Sw‐5b), AY007367 (Sw‐5c, Sw‐5d and Sw‐5e) and EF647603 (Sw‐5a^S^, Sw‐5* and Sw‐5**). Only the *Sw‐5* gene sequences from *S. lycopersicum* Heinz are shown in Table S2. *This gene has been reported previously and referred to as *Sw‐5f* (Rehman *et al*., 2009). However, this is not a paralogue of *Sw‐5b*, but an orthologue from *S. lycopersicum* as well as *Sw‐5a^S^*. **This gene is also from *S. lycopersicum*, being the highest conserved orthologue of *Sw‐5d* from *S. peruvianum*.

## References

[mpp12439-bib-0001] Ade, J. , DeYoung, B.J. , Golstein, C. and Innes, R.W. (2007) Indirect activation of a plant nucleotide binding site‐leucine‐rich repeat protein by a bacterial protease. Proc. Natl. Acad. Sci USA, 104, 2531–2536. 17277084 10.1073/pnas.0608779104 PMC1790868

[mpp12439-bib-0002] Andolfo, G. , Jupe, F. , Witek, K. , Etherington, G.J. , Ercolano, M.R. and Jones, J.D. (2014) Defining the full tomato NB‐LRR resistance gene repertoire using genomic and cDNA RenSeq. BMC Plant Biol. 14, 120. 24885638 10.1186/1471-2229-14-120 PMC4036795

[mpp12439-bib-0003] Berger, P.H. , Hunt, A.G. , Domier, L.L. , Hellmann, G.M. , Stram, Y. , Thornbury, D.W. and Pirone, T.P. (1989) Expression in transgenic plants of a viral gene product that mediates insect transmission of potyviruses. Proc. Natl. Acad. Sci USA, 86, 8402–8406. 2813397 10.1073/pnas.86.21.8402 PMC298290

[mpp12439-bib-0004] Bernoux, M. , Ve, T. , Williams, S. , Warren, C. , Hatters, D. , Valkov, E. , Zhang, X. , Ellis, J.G. , Kobe, B. and Dodds, P.N. (2011) Structural and functional analysis of a plant resistance protein TIR domain reveals interfaces for self‐association, signaling, and autoregulation. Cell Host Microbe, 9, 200–211. 21402359 10.1016/j.chom.2011.02.009 PMC3142617

[mpp12439-bib-0005] van der Biezen, E.A. and Jones, J.D. (1998) The NB‐ARC domain: a novel signalling motif shared by plant resistance gene products and regulators of cell death in animals. Curr. Biol. 8, 226–227. 10.1016/s0960-9822(98)70145-9 9545207

[mpp12439-bib-0006] Boiteux, L.S. (1995) Allelic relationships between genes for resistance to tomato spotted wilt tospovirus in *Capsicum chinense* . Theor. Appl. Genet. 90, 146–149. 24173797 10.1007/BF00221009

[mpp12439-bib-0007] Brommonschenkel, S.H. , Frary, A. , Frary, A. and Tanksley, S.D. (2000) The broad‐spectrum tospovirus resistance gene Sw‐5 of tomato is a homolog of the root‐knot nematode resistance gene Mi. Mol. Plant–Microbe Interact. 13, 1130–1138. 11043474 10.1094/MPMI.2000.13.10.1130

[mpp12439-bib-0008] Bucher, E. , Sijen, T. , De Haan, P. , Goldbach, R. and Prins, M. (2003) Negative‐strand tospoviruses and tenuiviruses carry a gene for a suppressor of gene silencing at analogous genomic positions. J. Virol. 77, 1329–1336. 12502849 10.1128/JVI.77.2.1329-1336.2003 PMC140852

[mpp12439-bib-0009] Coll, N.S. , Epple, P. and Dangl, J.L. (2011) Programmed cell death in the plant immune system. Cell Death Differ. 18, 1247–1256. 21475301 10.1038/cdd.2011.37 PMC3172094

[mpp12439-bib-0010] Dangl, J.L. and Jones, J.D. (2001) Plant pathogens and integrated defence responses to infection. Nature, 411, 826–833. 11459065 10.1038/35081161

[mpp12439-bib-0011] Day, B. , Dahlbeck, D. , Huang, J. , Chisholm, S.T. , Li, D. and Staskawicz, B.J. (2005) Molecular basis for the RIN4 negative regulation of RPS2 disease resistance. Plant Cell, 17, 1292–1305. 15749765 10.1105/tpc.104.030163 PMC1088003

[mpp12439-bib-0012] Fenyk, S. , Townsend, P.D. , Dixon, C.H. , Spies, G.B. , San Eustaquio Campillo, A. , Slootweg, E.J. , Westerhof, L.B. , Gawehns, F.K.K. , Knight, M.R. , Sharples, G.J. , Goverse, A. , Palsson, L.O. , Takken, F.L.W. and Cann, M.J. (2015) The potato nucleotide‐binding leucine‐rich repeat (NLR) immune receptor Rx1 is a pathogen‐dependent DNA‐deforming protein. J. Biol. Chem. 290, 24 945–24 960. 10.1074/jbc.M115.672121 PMC4599002 26306038

[mpp12439-bib-0013] Gabriels, S.H.E.J. , Vossen, J.H. , Ekengren, S.K. , van Ooijen, G. , Abd‐El‐Haliem, A.M. , van der Berg, C.M. , Rainey, D.Y. , Martin, G.B. , Takken, F.L.W. , de Wit, P.J.G.M. and Joosten, M.H.A.J. (2007) An NB‐LRR protein required for HR signalling mediated by both extra‐ and intracellular resistance proteins. Plant J. 50, 14–28. 17346268 10.1111/j.1365-313X.2007.03027.x

[mpp12439-bib-0014] Gaj, T. , Gersbach, C.A. and Barbas, C.F. (2013) ZFN, TALEN, and CRISPR/Cas‐based methods for genome engineering. Trends Biotechnol. 31, 397–405. 23664777 10.1016/j.tibtech.2013.04.004 PMC3694601

[mpp12439-bib-0015] Green, M.R. and Sambrook, J. (2012) Molecular Cloning: A Laboratory Manual. Cold Spring Harbor, NY: Cold Spring Harbor Laboratory Press.

[mpp12439-bib-0016] Hallwass, M. , de Oliveira, A.S. , de Campos Dianese, E. , Lohuis, D. , Boiteux, L.S. , Inoue‐Nagata, A.K. , Resende, R.O. and Kormelink, R. (2014) The tomato spotted wilt virus cell‐to‐cell movement protein (NSm) triggers a hypersensitive response in Sw‐5‐containing resistant tomato lines and in *Nicotiana benthamiana* transformed with the functional Sw‐5b resistance gene copy. Mol. Plant Pathol. 15, 871–880. 24720811 10.1111/mpp.12144 PMC6638845

[mpp12439-bib-0017] Heidrich, K. , Wirthmueller, L. , Tasset, C. , Pouzet, C. , Deslandes, L. and Parker, J.E. (2011) Arabidopsis EDS1 connects pathogen effector recognition to cell compartment‐specific immune responses. Science, 334, 1401–1404. 22158818 10.1126/science.1211641

[mpp12439-bib-0018] Fariselli, P. , Molinini, D. , Casadio, R. and Krogh, A. (2007) Prediction of Structurally‐Determined Coiled‐Coil Domains with Hidden Markov Models. In: Bioinformatics Research and Development (Hochreiter S. and Wagner R., eds), Pages 292–302, Berlin: Springer Heidelberg.

[mpp12439-bib-0019] van der Hoorn, R.A. and Kamoun, S. (2008) From guard to decoy: a new model for perception of plant pathogen effectors. Plant Cell, 20, 2009–2017. 18723576 10.1105/tpc.108.060194 PMC2553620

[mpp12439-bib-0020] Jahn, M. , Paran, I. , Hoffmann, K. , Radwanski, E.R. , Livingstone, K.D. , Grube, R.C. , Aftergoot, E. , Lapidot, M. and Moyer, J. (2000) Genetic mapping of the Tsw locus for resistance to the tospovirus tomato spotted wilt virus in *Capsicum* spp. and its relationship to the Sw‐5 gene for resistance to the same pathogen in tomato. Mol. Plant–Microbe Interact. 13, 673–682. 10830267 10.1094/MPMI.2000.13.6.673

[mpp12439-bib-0021] Karimi, M. , Inze, D. and Depicker, A. (2002) GATEWAY vectors for Agrobacterium‐mediated plant transformation. Trends Plant Sci. 7, 193–195. 11992820 10.1016/s1360-1385(02)02251-3

[mpp12439-bib-0022] Larkin, M.A. , Blackshields, G. , Brown, N.P. , Chenna, R. , McGettigan, P.A. , McWilliam, H. , Valentin, F. , Wallace, I.M. , Wilm, A. , Lopez, R. , Thompson, J.D. , Gibson, T.J. and Higgins, D.G. (2007) Clustal W and Clustal X version 2.0. Bioinformatics, 23, 2947–2948. 17846036 10.1093/bioinformatics/btm404

[mpp12439-bib-0023] Leipe, D.D. , Koonin, E.V. and Aravind, L. (2004) STAND, a class of P‐loop NTPases including animal and plant regulators of programmed cell death: multiple, complex domain architectures, unusual phyletic patterns, and evolution by horizontal gene transfer. J. Mol. Biol. 343, 1–28. 15381417 10.1016/j.jmb.2004.08.023

[mpp12439-bib-0024] Letunic, I. , Doerks, T. and Bork, P. (2012) SMART 7: recent updates to the protein domain annotation resource. Nucleic Acids Res. 40, 302–305. 10.1093/nar/gkr931 PMC3245027 22053084

[mpp12439-bib-0025] Lukasik‐Shreepaathy, E. , Slootweg, E. , Richter, H. , Goverse, A. , Cornelissen, B.J. and Takken, F.L. (2012) Dual regulatory roles of the extended N terminus for activation of the tomato MI‐1.2 resistance protein. Mol. Plant–Microbe Interact. 25, 1045–1057. 22512381 10.1094/MPMI-11-11-0302

[mpp12439-bib-0026] Maekawa, T. , Cheng, W. , Spiridon, L.N. , Toller, A. , Lukasik, E. , Saijo, Y. , Liu, P. , Shen, Q.H. , Micluta, M.A. , Somssich, I.E. , Takken, F.L. , Petrescu, A.J. , Chai, J. and Schulze‐Lefert, P. (2011) Coiled‐coil domain‐dependent homodimerization of intracellular barley immune receptors defines a minimal functional module for triggering cell death. Cell Host Microbe, 9, 187–199. 21402358 10.1016/j.chom.2011.02.008

[mpp12439-bib-0027] Maqbool, A. , Saitoh, H. , Franceschetti, M. , Stevenson, C.E.M. , Uemura, A. , Kanzaki, H. , Kamoun, S. , Terauchi, R. and Banfield, M.J. (2015) Structural basis of pathogen recognition by an integrated HMA domain in a plant NLR immune receptor. eLife, 4, e08709. 10.7554/eLife.08709 PMC4547098 26304198

[mpp12439-bib-0028] Medzhitov, R. (2001) Toll‐like receptors and innate immunity. Nat. Rev. Immunol. 1, 135–145. 11905821 10.1038/35100529

[mpp12439-bib-0029] Mestre, P. and Baulcombe, D.C. (2006) Elicitor‐mediated oligomerization of the tobacco N disease resistance protein. Plant Cell, 18, 491–501. 16387833 10.1105/tpc.105.037234 PMC1356554

[mpp12439-bib-0030] Meyers, B.C. , Kozik, A. , Griego, A. , Kuang, H. and Michelmore, R.W. (2003) Genome‐wide analysis of NBS‐LRR‐encoding genes in Arabidopsis. Plant Cell, 15, 809–834. 12671079 10.1105/tpc.009308 PMC152331

[mpp12439-bib-0031] Mucyn, T.S. , Clemente, A. , Andriotis, V.M.E. , Balmuth, A.L. , Oldroyd, G.E.D. , Staskawicz, B.J. and Rathjen, J.P. (2006) The tomato NBARC‐LRR protein Prf interacts with Pto kinase in vivo to regulate specific plant Immunity. Plant Cell, 18, 2792–2806. 17028203 10.1105/tpc.106.044016 PMC1626632

[mpp12439-bib-0032] Pappu, H.R. , Jones, R.A. and Jain, R.K. (2009) Global status of tospovirus epidemics in diverse cropping systems: successes achieved and challenges ahead. Virus Res. 141, 219–236. 19189852 10.1016/j.virusres.2009.01.009

[mpp12439-bib-0033] Peiro, A. , Canizares, M.C. , Rubio, L. , Lopez, C. , Moriones, E. , Aramburu, J. and Sanchez‐Navarro, J. (2014) The movement protein (NSm) of tomato spotted wilt virus is the avirulence determinant in the tomato Sw‐5 gene‐based resistance. Mol. Plant Pathol. 15, 802–813. 24690181 10.1111/mpp.12142 PMC6638753

[mpp12439-bib-0034] Peyret, H. and Lomonossoff, G.P. (2013) The pEAQ vector series: the easy and quick way to produce recombinant proteins in plants. Plant Mol. Biol. 83, 51–58. 23479085 10.1007/s11103-013-0036-1

[mpp12439-bib-0135] Qi, D. and Innes, R.W. (2013) Recent Advances in Plant NLR Structure, Function, Localization, and Signaling. Frontiers In immunology. 4, 348. 10.3389/fimmu.2013.00348 PMC3801107 24155748

[mpp12439-bib-0035] Rairdan, G.J. and Moffett, P. (2006) Distinct domains in the ARC region of the potato resistance protein Rx mediate LRR binding and inhibition of activation. Plant Cell, 18, 2082–2093. 16844906 10.1105/tpc.106.042747 PMC1533967

[mpp12439-bib-0036] Rairdan, G.J. , Collier, S.M. , Sacco, M.A. , Baldwin, T.T. , Boettrich, T. and Moffett, P. (2008) The coiled‐coil and nucleotide binding domains of the potato Rx disease resistance protein function in pathogen recognition and signaling. Plant Cell, 20, 739–751. 18344282 10.1105/tpc.107.056036 PMC2329922

[mpp12439-bib-0037] Rehman, S. , Postma, W. , Tytgat, T. , Prins, P. , Qin, L. , Overmars, H. , Vossen, J. , Spiridon, L.N. , Petrescu, A.J. , Goverse, A. , Bakker, J. and Smant, G. (2009) A secreted SPRY domain‐containing protein (SPRYSEC) from the plant‐parasitic nematode *Globodera rostochiensis* interacts with a CC‐NB‐LRR protein from a susceptible tomato. Mol. Plant–Microbe Interact. 22, 330–340. 19245327 10.1094/MPMI-22-3-0330

[mpp12439-bib-0038] Robert, X. and Gouet, P. (2014) Deciphering key features in protein structures with the new ENDscript server. Nucleic Acids Res. 42, 320–324. 10.1093/nar/gku316 PMC4086106 24753421

[mpp12439-bib-0039] de Ronde, D. , Butterbach, P. , Lohuis, D. , Hedil, M. , van Lent, J.W. and Kormelink, R. (2013) Tsw gene‐based resistance is triggered by a functional RNA silencing suppressor protein of the tomato spotted wilt virus. Mol. Plant Pathol. 14, 405–415. 23360130 10.1111/mpp.12016 PMC6638720

[mpp12439-bib-0040] de Ronde, D. , Butterbach, P. and Kormelink, R. (2014a) Dominant resistance against plant viruses. Front. Plant Sci. 5, 307. 25018765 10.3389/fpls.2014.00307 PMC4073217

[mpp12439-bib-0041] de Ronde, D. , Pasquier, A. , Ying, S. , Butterbach, P. , Lohuis, D. and Kormelink, R. (2014b) Analysis of tomato spotted wilt virus NSs protein indicates the importance of the N‐terminal domain for avirulence and RNA silencing suppression. Mol. Plant Pathol. 15, 185–195. 24103150 10.1111/mpp.12082 PMC6638762

[mpp12439-bib-0042] Sato, S. , Tabata, S. , Hirakawa, H. , Asamizu, E. , Shirasawa, K. , Isobe, S. , Kaneko, T. , Nakamura, Y. , Shibata, D. , Aoki, K. , Egholm, M. , Knight, J. *et al* (2012) The tomato genome sequence provides insights into fleshy fruit evolution. Nature, 485, 635–641. 22660326 10.1038/nature11119 PMC3378239

[mpp12439-bib-0043] Sato, Y. , Ando, S. and Takahashi, H. (2014) Role of intron‐mediated enhancement on accumulation of an Arabidopsis NB‐LRR class R‐protein that confers resistance to cucumber mosaic virus. PLoS One, 9, e99041. 24915153 10.1371/journal.pone.0099041 PMC4051679

[mpp12439-bib-0044] Scholthof, K.B. , Adkins, S. , Czosnek, H. , Palukaitis, P. , Jacquot, E. , Hohn, T. , Hohn, B. , Saunders, K. , Candresse, T. , Ahlquist, P. , Hemenway, C. and Foster, G.D. (2011) Top 10 plant viruses in molecular plant pathology. Mol. Plant Pathol. 12, 938–954. 22017770 10.1111/j.1364-3703.2011.00752.x PMC6640423

[mpp12439-bib-0045] Slootweg, E.J. , Spiridon, L.N. , Roosien, J. , Butterbach, P. , Pomp, R. , Westerhof, L. , Wilbers, R. , Bakker, E. , Bakker, J. , Petrescu, A.J. , Smant, G. and Goverse, A. (2013) Structural determinants at the interface of the ARC2 and leucine‐rich repeat domains control the activation of the plant immune receptors Rx1 and Gpa2. Plant Physiol. 162, 1510–1528. 23660837 10.1104/pp.113.218842 PMC3707565

[mpp12439-bib-0046] Soosaar, J.L. , Burch‐Smith, T.M. and Dinesh‐Kumar, S.P. (2005) Mechanisms of plant resistance to viruses. Nat. Rev. Microbiol. 3, 789–798. 16132037 10.1038/nrmicro1239

[mpp12439-bib-0047] Spassova, M.I. , Prins, T.W. , Folkertsma, R.T. , Klein‐Lankhorst, R.M. , Hille, J. , Goldbach, R.W. and Prins, M. (2001) The tomato gene Sw5 is a member of the coiled coil, nucleotide binding, leucine‐rich repeat class of plant resistance genes and confers resistance to TSWV in tobacco. Mol. Breed. 7, 151–161.

[mpp12439-bib-0048] Sun, Q. , Collins, N.C. , Ayliffe, M. , Smith, S.M. , Drake, J. , Pryor, T. and Hulbert, S.H. (2001) Recombination between paralogues at the Rp1 rust resistance locus in maize. Genetics, 158, 423–438. 11333250 10.1093/genetics/158.1.423 PMC1461629

[mpp12439-bib-0049] Takahashi, H. , Suzuki, M. , Natsuaki, K. , Shigyo, T. , Hino, K. , Teraoka, T. , Hosokawa, D. and Ehara, Y. (2001) Mapping the virus and host genes involved in the resistance response in cucumber mosaic virus‐infected *Arabidopsis thaliana* . Plant Cell Physiol. 42, 340–347. 11266586 10.1093/pcp/pce039

[mpp12439-bib-0050] Takken, F.L. and Goverse, A. (2012) How to build a pathogen detector: structural basis of NB‐LRR function. Curr. Opin. Plant Biol. 15, 375–384. 22658703 10.1016/j.pbi.2012.05.001

[mpp12439-bib-0051] Tang, X. , Xie, M. , Kim, Y.J. , Zhou, J. , Klessig, D.F. and Martin, G.B. (1999) Overexpression of Pto activates defense responses and confers broad resistance. Plant Cell, 11, 15–29. 9878629 10.1105/tpc.11.1.15 PMC144088

[mpp12439-bib-0052] Van Ooijen, G. , Mayr, G. , Kaslem, M.M.A. , Albrecht, M. , Cornelissen, J.C. and Takken, F.L.W. (2008) Structure–function analysis of the NB‐ARC domain of plant disease resistance proteins. J. Exp. Bot. 59, 1383–1397. 18390848 10.1093/jxb/ern045

[mpp12439-bib-0053] van der Vossen, E.A. , van der Voort, J.N. , Kanyuka, K. , Bendahmane, A. , Sandbrink, H. , Baulcombe, D.C. , Bakker, J. , Stiekema, W.J. and Klein‐Lankhorst, R.M. (2000) Homologues of a single resistance‐gene cluster in potato confer resistance to distinct pathogens: a virus and a nematode. Plant J. 23, 567–576. 10972883 10.1046/j.1365-313x.2000.00814.x

[mpp12439-bib-0054] Wang, G.F. and Balint‐Kurti, P.J. (2015) Cytoplasmic and nuclear localizations are important for the hypersensitive response conferred by maize autoactive Rp1‐D21 protein. Mol. Plant–Microbe Interact. 28, 1023–1031. 26039083 10.1094/MPMI-01-15-0014-R

[mpp12439-bib-0055] Wang, G.F. , Ji, J. , Ei‐Kasmi, F. , Dangl, J.L. , Johal, G. and Balint‐Kurti, P.J. (2015) Correction: molecular and functional analyses of a maize autoactive NB‐LRR protein identify precise structural requirements for activity. PLoS Pathog. 11, e1004830. 25719542 10.1371/journal.ppat.1004674 PMC4342346

[mpp12439-bib-0056] Wang, R. and Brattain, M.G. (2007) The maximal size of protein to diffuse through the nuclear pore is larger than 60 kDa. FEBS Lett. 58, 3164–3170. 10.1016/j.febslet.2007.05.082 PMC4064367 17588566

[mpp12439-bib-0057] Wen, R.H. , Khatabi, B. , Ashfield, T. , Saghai Maroof, M.A. and Hajimorad, M.R. (2013) The HC‐pro and P3 cistrons of an avirulent soybean mosaic virus are recognized by different resistance genes at the complex Rsv1 locus. Mol. Plant–Microbe Interact. 26, 203–215. 23051173 10.1094/MPMI-06-12-0156-R

[mpp12439-bib-0058] Whitham, S. , Dinesh‐Kumar, S.P. , Choi, D. , Hehl, R. , Corr, C. and Baker, B.J. (1994) The product of the tobacco mosaic virus resistance gene N: similarity to toll and the interleukin‐1 receptor. Cell, 78, 1101–1115. 7923359 10.1016/0092-8674(94)90283-6

[mpp12439-bib-0059] Zhang, Y. , Dorey, S. , Swiderski, M. and Jones, J.D. (2004) Expression of RPS4 in tobacco induces an AvrRps4‐independent HR that requires EDS1, SGT1 and HSP90. Plant J. 40, 213–224. 15447648 10.1111/j.1365-313X.2004.02201.x

