# Supplementary material for: Cell death triggering and effector recognition by Sw‐5 SD‐CNL proteins from resistant and susceptible tomato isolines to Tomato spotted wilt virus
Source: Mol Plant Pathol. 2016 Aug 14;17(9):1442–54. doi: 10.1111/mpp.12439 (PMC6638320; doi:10.1111/mpp.12439)
Supplement: Supplementary file 1 — Table S1. Primers and vectors used for building and expression of the full, truncated and mutated Sw‐5 gene versions. [file MPP-17-1442-s001.docx]

| Gene/Construct | Primers sequences | | Entry vector | | Destination vectors | |
| --- | --- | --- | --- | --- | --- | --- |
| Full *Sw-5a, Sw-5b*, and *Sw-5a^S^* genes and their mutated versions after overlapping and fusion PCRs | Sw5b-pD1:  GGGGACAAGTTTGTACAAAAAAGCAGGCTTCATGGCTGAAAATGAAA TTGAGG  Sw5b-pD2:  GGGACCACTTTGTACAAGAAAGCTGGGTCCTAATCTGAGCGTTGTTTGACGAGG | | pDONR207 | | pK2GW7, pK7WGF2, pEAQ-DEST1 – and -DEST2 | |
| *Sw-5a-, Sw-5b-, Sw-5a^S^-*NB-ARC domains and their mutated versions after overlapping PCR | NB-SW5b-F: GGGGACAAGTTTGTACAAAAAAGCAGGCTTCGAAGGAGATAGAACCATGGCTGCTCCATTAAAACATCT  NB-SW5b-R:  GGGGACCACTTTGTACAAGAAAGCTGGGTCCTAAAGCATAAACTTTGCTTCTC | | pDONR207 | | pK2GW7, pK7WGF2, pEAQ-DEST1 – and -DEST2 | |
| *Sw-5b* CC domain | SW5N-ALL-F:  CCATGGTCATGGCTGAAAATGAAATTGAGG  CC-SW5N-R:  GCGGCCGCTCACACATAGTGAGGATTAAGAGG | | pENTR11 | | pK2GW7, pK7WGF2, pEAQ-DEST1 – and -DEST2 | |
| *Sw-5b* CC-NB-ARC domain | SW5N-ALL-F:  CCATGGTCATGGCTGAAAATGAAATTGAGG  CC-NB-ARC-SW5N-R:  GCGGCCGCTCAAAGCATAAACTTTGCTTCTC | | pENTR11 | | pK2GW7, pK7WGF2, pEAQ-DEST1 – and -DEST2 | |
| *Sw-5b* NB-ARC-LRR domain | NB-ARC-LRR-SW5N-F:  CCATGGTCGCTGCTCCATTAAAACATCTGC  SW5N-ALL-R:  GCGGCCGCTCAATCTGAGCGTTGTTTGAC | | pENTR11 | | pK2GW7, pK7WGF2, pEAQ-DEST1 – and -DEST2 | |
| *Sw-5b* LRR domain | LRR-SW5N-F:  CCATGGTCGCAGTGAAGGGTCAATATATCCA  SW5N-ALL-R:  GCGGCCGCTCAATCTGAGCGTTGTTTGAC | | pENTR11 | | pK2GW7, pK7WGF2, pEAQ-DEST1 – and -DEST2 | |
| *Q599R NB-ARC (*Sw-5a^S^*) | Sw5S-Q599R-1:  TCTCAAACGTATAATCGGAGAGAGTTATTA  Sw5S-Q599R-2:  AAAGATATCTTGTAATAACTCTCTCCGATT | | pDONR207 | | pEAQ-DEST2 | |
| ***F613S/N614D NB-ARC (*Sw-5a^S^*) | Sw5S-FN613SD-1:  AGTCAAGTTACAGGTTCCGACGACAATGGA  Sw5S-FN613SD-2:  GGAACCTGTAACTTGACTAAAGATATCTTG | | pDONR207 | | pEAQ-DEST2 | |
| *** V659D NB-ARC (*Sw-5a^S^*) | Sw5S-V659D-1:  GATGGAATTCGAAGCAGAATAGTCGTAACA  Sw5S-V659D-2:  TCTGCTTCGAATTCCATCATCTGGAAAAGA | | pDONR207 | | pEAQ-DEST2 | |
| **Q599R-*Sw-5a^S^* (CC-NB) + *Sw-5b* (LRR) | P2-Sw5S-CC-NB-R:  CTTCACTGCAAGCATAAACTTTGCTTC  P3-Sw5b-LRR-F:  GCAGTGAAGGGTCAATATATCCATTTTC | pDONR207 | | pK7WGF2 | |  |
| *Sw-5a^S^* CC | CC-pD-F:  GGGGACAAGTTTGTACAAAAAAGCAGGCTTCATGGCTGAAAATGAAATTGA  CC-pD-R:  GGGGACCACTTTGTACAAGAAAGCTGGGTCCTACACATAATGAGGATTAAGAG | | pDONR207 | | pK7WGF2 | |
| Q599R-*Sw-5a* NB-LRR | Sw-5aS NB-ARC-LRR  NB-LRR-pD-F:  GGGGACAAGTTTGTACAAAAAAGCAGGCTTCATGGCTGCTCCATTAAAACATCT  NB-LRR-pD-R:  GGGGACCACTTTGTACAAGAAAGCTGGGTCCTAATCTGAGCGTTGTTTGACGA | | pDONR207 | | pK7WGF2 | |

*Primers used for overlapping PCR (insertion of mutations);

**Primers used for fusion PCR (combined with primers with att sites also described above).
