# Supplementary material for: Cell death triggering and effector recognition by Sw‐5 SD‐CNL proteins from resistant and susceptible tomato isolines to Tomato spotted wilt virus
Source: Mol Plant Pathol. 2016 Aug 14;17(9):1442–54. doi: 10.1111/mpp.12439 (PMC6638320; doi:10.1111/mpp.12439)
Supplement: Supplementary file 2 — Table S2. Sw‐5 gene sequences from Solanum lycopersicum Heinz. [file MPP-17-1442-s002.docx]

| >Sw-5a^S^  atggctgaaaatgaaattgaggaaatgttagagcacctgagaaggatcaagagtggaggtgatctggattggttcgacatattgcgaattgaggaacttgaaatggtgctaagagtttttagaacctttacaaagtatcatgatgttcttttgcctgattccttagtcaaactcacaaagatggccaaattgactggggaaatacttcaccgggtgttgggtaggattccacataaatgtaaaactaaccttaatctagaaaggctagaatcacatttgttggaattctttcaaggtaatacggccagtttaagtcgcaattatgagttgaatgattttgatctgtcaaaatatatggattgtctggaaaaatttctaaatgatgtactgatgatgttcttgcaaaagggtaggtcctgccattccaaaagaaaacttgcaatacatcgatctataaaggaactgaaaattgttcaaaagaaaatgagatttttgaaatacatatatgccacagagataaatggttatgtcgactatgagaagcaggaatgtttggagattcgaattcagttcatgactaacactgtgggacaatattgtgtggccgtattagattatgtcgctgagggtgaacttaatgatgaaaatgacaactttagtaaacctccttacctattatcattgattgtgtttgtggagctggaaatgaagaagctttttcatggtgaagtaaaggcttcaaagtttactcgatcaaaaactttcaaggacaagaaattaccaaaaggattttcacatcatctccacaatctgttgatgtatctcagaaacaaaaagctcgagaattttcctaataatatcgctgctcaaaatatcgatgtggcaatagagttcttgttggttttccttgatgctgatgtgtcaaatcatgttattaatggtaactggttgaaagaggtcttgttaaaggttggagctatagcgggtgatattctatatgtaattcaaaagcttcttcctagatcaataaacaaagatgaaactagcaacataagtctttgctcaatacagatattggagaagactaaagatctgaaggcacaagttgagacgtactacaaatccttaaaatttactccatctcagttccccacctttggtggattgagctttctggattctcttttaaggaaactgaatgagatgtcgacatctaagtccggattaggtttcctgacgaaacctcttttagggaatttggagaaagagctatcatctcttgcatccattttagagaaggagctctcatccattttcagtgatgtcgtgcaccatgaacataacattcctaaagatcttcagagacgtaccatcaatttggcatgtgaggctgaggttgctattgattctattcttgctcagtataatgtttttttgcatattttttgctcacttcctacaattgtaaaagagatcaagcaaattaatgcagaggtgactgagatgtggtcagcggacattcctcttaatcctcattatgtggctgctccattaaaacatctgccggatcgacatagcaatcttgtaactgatgaggaggtagtgggttttgagaataaagcagaagaactaattgattatctgattagaggtacaaatgagctagacgttgtcccaattgtaggcatgggaggacaagggaaaacgacaattgctagaaagttgtacaataatgacattattgtttctcgctttgatgttcgagcatggtgcatcatttctcaaacgtataatcagagagagttattacaagatatttttagtcaagttacaggtttcaacgacaatggagctacggttgacgttcttgccgacatgttgaggagaaaattaatgggaaagagatatctcattgtattggatgatatgtgggattgtatggtatgggatgacttaaggctttcttttccagatgttggaattagaagcagaatagtcgtaacaactcgacttgaagaagtgggtaagcaagtcaagtaccatactgatccttattctcttccattcctcacaacagaagagagttgccaattgttgcagaaaaaagtgtttcaaaaggaagattgcccgcctgaactacaagatgtgagtcaagcagtagcagaaaaatgcaaaggactgcccctagtggttgtcttggtagctggaataatcaaaaaaaggaaaatggaagaatcttggtggaatgaggtgaaagatgctttatttgactatcttgacagtgagttcgaagaatatagtctggcaactatgcagttgagttttgataacttaccccactgtttaaagccttgtcttctttatatgggaatgttttcggaggacgcaagaattccagcatctacattgataagtttatggattgctgaaggattcgtggagaacactgaatctggtacattaatggaagaggaagctgaaggttacttgatggatctcattagcagtaacatggtaatgctttcaaagagaagttataagggtaaagtcaaatactgtcaggttcatgatgttgtgcatcacttttgcttggaaaagagtagagaagcaaagtttatgcttgcagtgaagggtcaatatatccagtttcaacctttggattggaagggaactcgagtgagcttcagttttagtgaagagctttccaagtttgcatctctggtctccaaaacacagaagcctttccatcaacacttgaggtcattgataacgaccaatcgagcagaatctattgatgtgattctcttctgtcagattagtgaattgcgacttcttaaagtcttggatttgagttcttatactgtggaatttttgtcgttagctacattcaaaccactaaatcagctgaagtacctcgcagttcaggctgataaattctattttgatccaggatcacatcttccccatatagaaactttcattgtaaagaattttccttatggtatagggttaccagtgtctttttgggaaatgaaaaaattaaggcatgctcattttggtaaggctgaatttgataagcaggggctctctgaaggatcctctaaattggaaaatttgaggatattaaagaatattatacaatttgatagggtggatgtgttatcaacgaggtgtcctaatcttcaacaacttcaaatcacatattttgggaataatgaagagcctttttgtcccaaattggagaatcttacccagcttcaacaacttcaactttcctttgtgcgtccccgcactctatccgggttacagttgccttcaaatttaaataagttggtacttgaaggaattcatatagaaagtgttattcccttcattgcgggactaccaagcctggaatatctccgattacaggatgtgtgttttcctcaatcagaagagtggtgccttggagatatgacgttccataaacttaagttgttgaaactggtgaagttaaatatatcaaggtgggatgtctcagaggaatcatttccgttgcttgaaacactcgttataaagaagtgcggtgacctagaggagatcccagttagctttgctgatattcccacattggaacagattaaattgattgggtcctggaaagtatctctggaggattcagctgtgagaatgaaggaagaaatcatacagattgaaggatgtgatcgtttacacctcgtcaaacaacactcagattga |
| --- |
| >Sw-5*  atgtcgctcttaaagaaactaattccaagagttggtacacttgcaggaagtcttgtacggaagatggctcaaaatgaaattgaagatatgttagatcacctgagaaggatcaagagtggaggtaatctggatagggtcaagatcaatcgaattgagaaacttgaaatggtgttcaagtatcatcatgttcttttgcctgattccatcgtcaaactcaaaaagaaggacaaatggattgcgaaaatggttcagtgggtattggatggaattccggatgaatgtaaaactaaccttaatttaagaaggttagaattacatttgttggaattctttgaaggtaaaatcagtttaagttacaaccatgagttgaatgattctgatctgtcgaaatatatggattgtgttggaaagaatctaaatgatgtactgatgattttactgcaaagggctaggtatgaccctccaggagaaaacctcgaaatccacagatttataaagcaattaaaaattgttcaaaagaaaatgagatttttgagatacttatatgttatagagataaatggttacgtcaaccaagagaagttggaatgtttggacactcgaatgcattttatggcttacaatgtgggacaactttgtcttgctattttaggttacgatatttctaattttgtagatgatgatgatgatgatgatgatgatgatgatgatgatgatgatgatgatgatgatgaggatgaggatggggatggggatggggatggggatgatatcttgaataaacctccttatttattattcttgattatcttagtagagctggaaatgaagaaaatttttctcggtgaactaaaggcttccaagtttactcaatcaagaactttcaaggacaagaaattaccaaaaggattttctcatcatctccatagtctgttgatgtatcttagaaacaaaaagcttgagaaatttcctaataataactctcctcaaaatattgatatagcaatagagttcttgttggttttccttgatgctgatgtcggaaatcatgtaattaatggtaactggttgaatgaagttatggaaaaggttggagctatagcgggtgatgttctatatgtaattcaaaagcttcttcctagctctataaaaaaagatgacaatagcaaaataagtctttgctcaatacagatatgggagaaaactaaagatctgaaggcacaagtggagacgcactataaatccttaaaattcactccatcgcaattccccactgttggtggatggagctttctggattctcttatgcggaaactgaaagagatgtcgaaatctaaatcttgtttagatttcctgatgaaacctcttttggggaatttggagaaagagctatcagctcttacatccattttagagaaggatctgtcatctttatcatccattttaagcgatgtcgccaaggtgcaccatgaacatgaaattcttcaagatcttcacaggcgtactatcaattgggcatatgaagcggaagttgcgattgactctattcttgctcagtataatgtcttttggcatattttttgctcacttcctacaatcttaaaagaaatcaagcaaattaatgtgcaggtgactcagatgtggtcagcggacattgctcttaagccttgctatatggtagcaccatttgaatacttcccaactcgacatatcaatccagtgactgatgaggatatagtgggttttgggaatgacatagaaaaaatgtttcagtatctgattagaggtacaaatgatctagacgttgtcccaattgtaggcatgggtggacaagggaaaacgacagttgctagaaaggtgtacaatagtgacaacattgtttctcattttgatgttcgagcatggtgcatcgtttcccaaacatataaccggagaaagctattacaagacattttgagtcaagttaccggttccaaggacaaggggtatgaggatgatatccttgctgatgagttgaggaaaagcttaatgggcaagagatatctcattgtcttggatgatatgtgggattgtatggcatgggatgacttaaggctttcctttccagattccggaaatagaagcagaatagtagtaacaactcgacttgagaaagtgggcgaacaagtcaagtgccatactgatccttattctcttccgttcctcacaaaagaagagagttgtgaattgttgcagaaaaaagtgtttcaaaaggaagatttcccgcctgaactacaagatgtgagtcgagcagttgcagaaaaatgcaaaggactgcccctagtggttgtcttggtagctggaataatcaaaaaaaggaaaatggaagaatcttggtggaatgaggtgaaagattctttatttgaccaccttaatcgtgagtcggaagaatatagtctttcaactatgcagttgagttactataacttaaccgactgtttaaagccttgtcttctttatatggggatgtttcaggaggatgcaataattcgagtatctgaattgataagtctatggattgcggaaggatttgtgcagagcattgaatctgggagattgttgatggaagaggcagctgaaggttacttgatggatctcattcagagtaacgtggtaatggtttcaaatagaaggtataatggtaaggtcaaatactgtcaggttcatgacgtagtgcttcacttttgcttggagaagagtctagaagaaaagtttatgttggcagtgaaggggcatgatagccagtttcaaccttttgaatggaaggaaaaccgagtgagcttcagtttcagtaaagagctttccaagtttgcatctctaggatccaaaacacggaagtctttccaccaacacttgaggtcactgataacgataaataacctagagttaattgatgggattcccttctgtcagattcttaaattgcgacttcttaaggtcttgattttgacttcccatgaagtggattatttgtcgtcagctacattcaaacctctaaatcacctgaaatacctcgcagtttgtgcaaataagttttactttcatccagaatcacatctgcccaatcttgaaactttaatcgtaaataattgggaaaatgaagtagagttaccagcgtctttttgggagatggaaaaattaaggcatgttaagatttgggatgctaaatttgatttggaagaggataatcagggatcctccgaattggaaaatttgaggatattacagtatgtaaaatttccaattgatggaagggatagggtggatatgttatcaaggaggtgtccaaatcttcaaaaactgcacatcaacttggaatatcggggcagtaataattcggcagatttattttgtctcacattagagaatcttactcagcttcaaaatcttcgcctaactgttgagaggtccaacattgtatctgggttacaattgccttcaaatttaaacaagttggtactaagtggggctcatatcgaaaacctgagttccttcattgcaggactaccaagcctggagtatctccaattttgtgacccctatgaatctgttcaaatcagagattggtgccttggaggtatcacgttccctaaacttaagttgttgaaactggtgaacttaccgatctcaaggtgggatgcctcagaggaatcgtttccccagctcgaaacacttgttataaaaaggtgtaaccacctcaaggagatcccccttagctttgctgatattccaacactgaagcagattaagttgattaggtgcgagaacgaatctctcaaggattcagctgtggagattaagaaagacgttgaagagaatgaaggaaacgaccgtattgacctcattatcaaagtaagtagaaacaaactcctaaatatgttctgtttttttgagtatctaactaacttgacaccactatgtataatacagggatggtag |
| >Sw-5**  atggctcaaaatgaaattgaggaaatgttagatcgcttgcgaaggatcgagagtagaggtaatctgagtagcgtcgagattgggaaagttgagaaatttgaaagaatgctaagatttcttagaaccattacaagttatcatcgttttattttccctgatacattagtcaaactcacaaagaaggccaaatcgattgtgaaaacacttcaaggaatatttgatgggattccagatgaatgtaaaactaaccttaatctggaaaggctcgtatcacatttgttcgaattctttcaaggtaatacgaccagtttaagttgcaattctgagtcgaatgattttgatctctcagaatgtatggattggctcgtaaagaatataaatgatgtactgattctcgtgggacagcctatggatgactcatcttgtcggtctaggttagagtttctatttataaagcaactaaaaattattcacaagaaaatgagatttttgagatacttatatgccacagaaataaatggttacattgactatgagaagttggaaggtctggagacccgaattcaggtcttggctgataatgtaggacaattatgtactgtatttattgtactcgaggatgaggatgaggatgaggatgacaatgaaatagttgatatagttgagaataaacctccttatctattagggttgattgtgttagtggagcaggaaatgaagaagatttttcttagtgaattaaaggcttcaaagttcacccaatcaaaaacattcaaggacaagaaattaccaaaaggattttcacatcatctcaacagtctgttggtgtatctcagaaacacaaagctcaagaactttcctgataatgtgtctgctcaaaatattggtctgtcaatagagcttctgttgttttttcttgatgctgatctttcaaatcatgttattagtcgaaactggttgaatgagatcttggaaaacgtggcaaccatagcgggtgatgttctatacgtaattcaaaagcttcttcctagatcaataagcaaagacgatgacactagcaagataaacatttactcgatgcaggtattggagaaaactaaagatctgaaggcacaagtagagacgtactacaaatccttaatatttactccatctcagttccctacctctggtggtttgacctttatggattctcttttaaggaaattaaatgagatgtcaagatctaaatatggtttagatttgctgatgaaacctatttactgtaatttggagaaagagcaatcaactcttatatccaatttagagaaggagttgtcatctttatcatccattttcagagatgtcacaaaggtgcaccatgaacataaagatcttcaaagacgtaccatcaatttggcatatgaagctggggtttccattgactctatttgtgctaaatataatgttttttggcattgtttttgctcacttccaacaatcgtagaagagatcaagcaaattaatgcagaggtaaccgagatgtggtcagcggacattcctcttaatcctcactatgtggctgcaccatttaaacacctgccaactcgtcatagcaatcccattactgatgaggagatagtgggttttgggattgacaaagaaaaaataattcagtgtctgattagaggtacaaatgatctagacgttgtcccaattgtaggcatggggggacaagggaaaaccacaattgctagaaaggtgtacaatagtgacaacattgtttctcattttgatgttcgagcatggtgcatcgtttcccaaacatataaccggagaaagctattacaagagattttgagtcaagttaccggttccaaggacaagggatatgaggatgatatccttgctgatgagttgaggaaaagcttaatgggaaagagatatctcattgtcttggatgatatgtgggattgtatggcatgggatgacttaaggctttcctttccagattttggaaatagaagcagaatagtagtaacaactcgacttgagaaagtgggcgaacaagtcaagttccatactgatccttattctcttccattcctcacaaaagaagagagttgcaaattgttgcagaaaaaagtttttcaaaaggaagattgtccgcctgaactacaagatgtaagtcaagtagttgcagaaaaatgcaagggactgcctctagtgattgtcttggtagctggaataatcaaaacaaggaaactggaagaatcttggtggaatgaggtgaaagattctttatttgactatcttgattgtcattcagagcaatatagtcgggctactatgcagttgagttttgataacttagctgattgtttaaaaccttgtcttctttatatggggatgtttccggaggatgcaagcattaacgtgtctgcattgttaagtttatggttagcggaagacttcgttcagaacattaaatctgctgaagattacttgatgaatctcattagcagtaacgtggtaatggtttcaaagaaagaatataatggcaagatcaaatactgtgaggttcatgatgtagtgcttcacttttgcttggagaagagtagagaagaaaattttatgttggttgcgaagggaaatcatagtcaatttcaatcttttgtttggaagcaaagtcgagtgagcttcaatttgattgaagagaattccaagtttgcatccaaaacaaggaagtgttcccatcaaccgttgaggtcactgataacgaacggagcatctttttatataatgtccttaagttcttggattcataatttgcgacttcttaaggtcttggttttgagttcctatgaagttgactatgtgaattcagctacactgaaaccacttaattacctgaagtacctcggagtaagggcaactacgttctattttgatcgagaatcacatctgccccatcttgaaactttaattgtgaagaatgatagatcagttatgttacgagggtgtttttgggaaatggaacaattaaggcatgttgagatcaatgatgctgaatttgataagcaggggctctttgaaggatcctctaaattggaaaatttgaggatattaaagaacattgttggatttccaattgatagggcggatgtgttatcaaggaggtgtcctaatcttcaacaacttcaaatcgaatttggatcttttgcagaacctctttggctcacattggagaatcttacccagcttcaaatacttgacctttcctttaggttgttccacatgctatctgggttacaattgccttcaaatttaaagatgttggtactaagagacgctgatctagaaaacctaacttccttctttgctggactaccaagcctggagtatcttcaattaacaaatatgtattttcctcaatcagaatggtgccttggagatatcacgtttcataaactcaagttgttgaaactggtgcagttagatatctcaaggtgggatgtctcggaggaatcctttcccctgcttgaaacaatcgttataagatggtgccataacctcgaggaaatccccattagctttgcagatgttccaacactgaaacagattaagttggttcactgcaagaacaaatctctggaggattcagctgtgaggattaagaaagatgttgaagagaatgaaggaaacgaccgtattgacctcattatcaaagtaagtagaaataaactccgatgttttgtttttgagtatttacctgcatctaacttgacaccaataatacagaaatag |
