# Supplementary figures and images for: Cell death triggering and effector recognition by Sw‐5 SD‐CNL proteins from resistant and susceptible tomato isolines to Tomato spotted wilt virus
Source: Mol Plant Pathol. 2016 Aug 14;17(9):1442–54. doi: 10.1111/mpp.12439 (PMC6638320; doi:10.1111/mpp.12439)

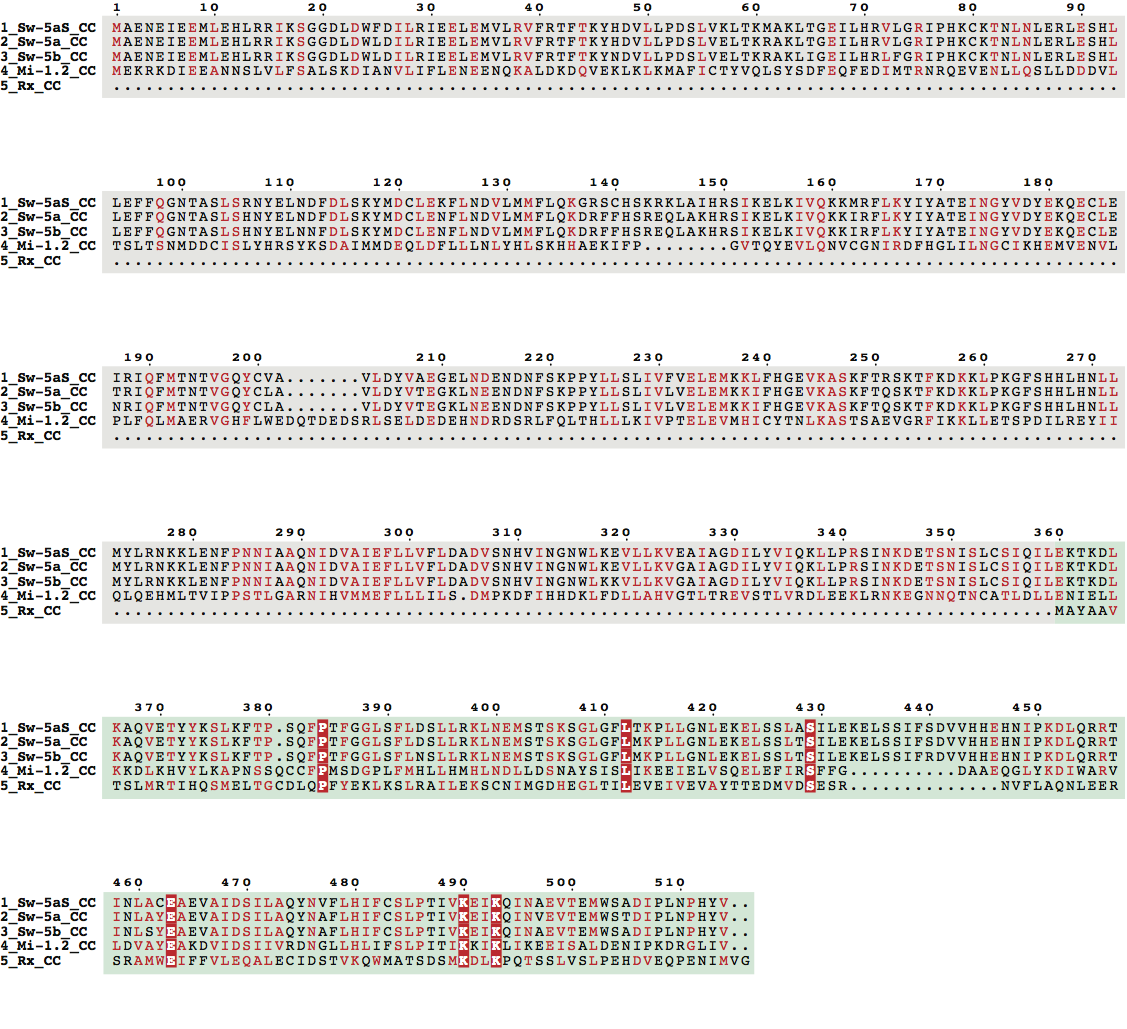

Supplement: Supplementary file 3 — Fig. S1. Multiple sequence alignment of SD‐CC domains from Sw‐5a (AY007366), Sw‐5b (AY007366), Sw‐5aS (Table S2), Mi‐1.2 (AF039682) and Rx (AJ011801). The amino acid residues shaded in green indicate a putative coiled‐coil (CC) domain in comparison with potato Rx, which lacks the so‐called Solanaceae domains (SD). The area shaded in grey covers the extended N‐terminus previously divided as the N‐terminal domain and SD for potato Prf (Mucyn et al., 2006) or SD1 and SD2 domains for tomato Mi‐1.2 (Lukasik‐Shreepaathy et al., 2012). [file MPP-17-1442-s003.tiff]

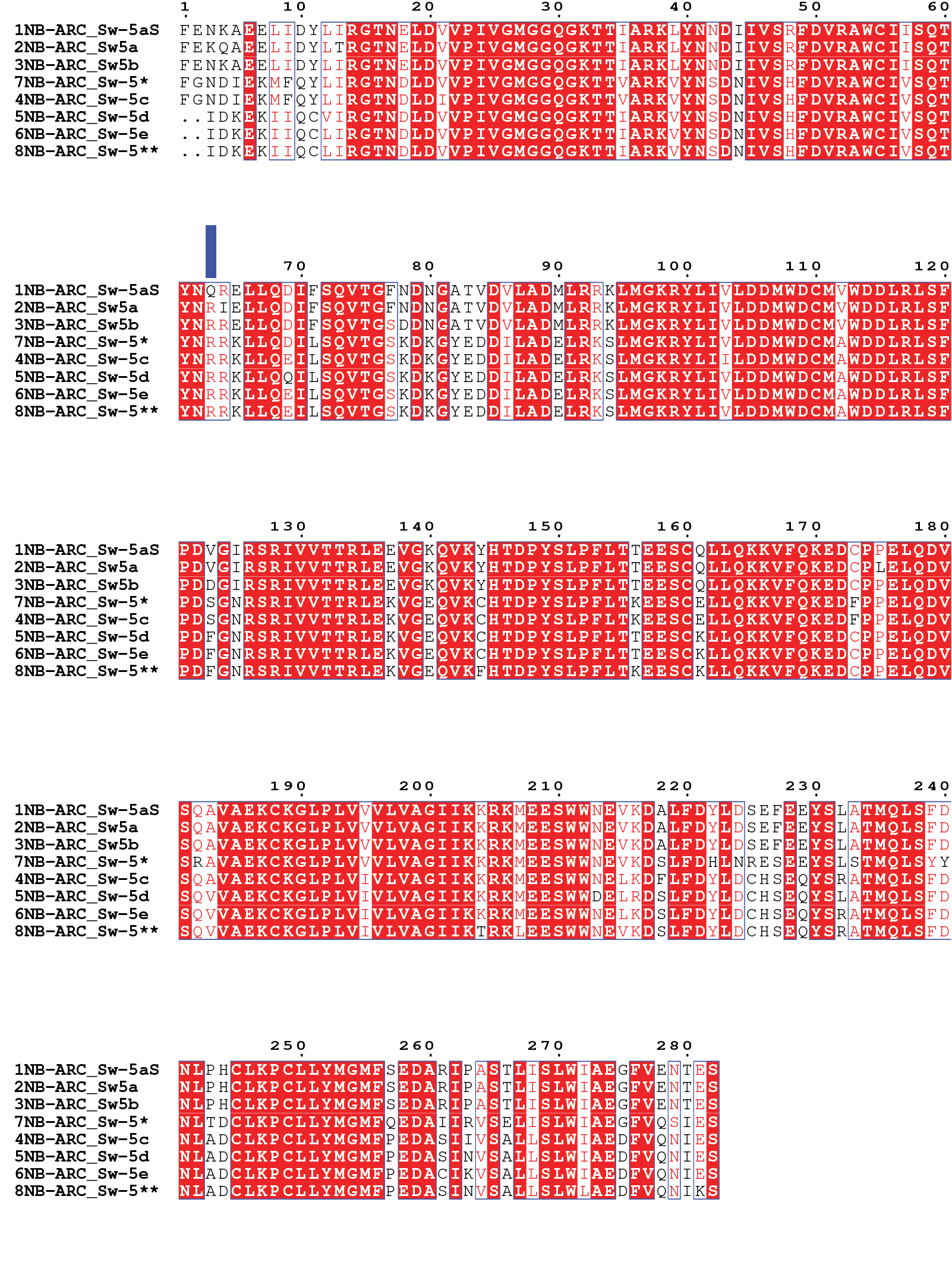

Supplement: Supplementary file 4 — Fig. S2. Multiple sequence alignment of NB‐ARC [nucleotide‐binding adaptor shared by Apaf‐1 (from humans), R proteins and CED‐4 (from nematodes)] domains from available and full Sw‐5 protein sequences from Solanum peruvianum and S. lycopersicum Heinz. The blue bar indicates the Q599R mutation. GenBank accessions: AY007366 (Sw‐5a and Sw‐5b), AY007367 (Sw‐5c, Sw‐5d and Sw‐5e) and EF647603 (Sw‐5aS, Sw‐5* and Sw‐5**). Only the Sw‐5 gene sequences from S. lycopersicum Heinz are shown in Table S2. *This gene has been reported previously and referred to as Sw‐5f (Rehman et al., 2009). However, this is not a paralogue of Sw‐5b, but an orthologue from S. lycopersicum as well as Sw‐5aS. **This gene is also from S. lycopersicum, being the highest conserved orthologue of Sw‐5d from S. peruvianum. [file MPP-17-1442-s004.tif]
